# An Exploration of Oral-Gut Pathogens Mediating Immune Escape of Pancreatic Cancer *via* miR-21/PTEN Axis

**DOI:** 10.3389/fmicb.2022.928846

**Published:** 2022-06-22

**Authors:** Rui Li, Yaoyuan Hu, Shuhong Hou

**Affiliations:** Department of General Surgery, Shengjing Hospital of China Medical University, Shenyang, China

**Keywords:** oral-gut pathogens, pancreatic cancer, metastasis, miR-21/PTEN axis, immune escape

## Abstract

Oral-gut pathogens are closely associated with pancreatic cancer, such as *Campylobacter jejuni, Clostridium difficile, Enterococcus faecalis, Escherichia coli, Fusobacterium nucleatum, Helicobacter pylori, Porphyromonas gingivalis*, and *Vibrio cholera*, but the related mechanisms remain not well understood. Phosphatase and tensin homolog (PTEN, a widely known tumor suppressor) play a key role in the anti-cancer immune system. Pancreatic cancer cells with PTEN loss are often in the immunosuppressive tumor microenvironment regulated by myeloid-derived suppressor cells (MDSCs), regulatory T cells (Tregs), and M2 macrophages, which are regarded as the mechanism in the immune escape of cancers. The miR-21, as an oncogene in human cancers, plays an important role in pancreatic cancer progression, downregulates the levels of PTEN, and may promote cancer to evade host immune surveillance. Some oral-gut pathogens have been found to promote miR-21 expression and reduce PTEN expression. On the other hand, most gut pathogens infection is thought to produce reactive oxygen species (ROS) or activate inflammatory cytokines, which may also induce ROS-mediated miR-21 expression. These pathogens' infection is involved with the cell density of MDSCs, Tregs, and M2 macrophages. Therefore, it is quite reasonable to propose that oral-gut pathogens possibly promote pancreatic cancer escaping from host immune surveillance by activating the miR-21/PTEN axis and immune-suppressive cells. The present exploration suggests that an increased understanding of the pattern of the effects of gut pathogens on the miR-21/PTEN axis will lead to better insights into the specific mechanisms associated with the immune escape of pancreatic cancer caused by oral-gut microbiota.

## Introduction

Pancreatic cancer is highly metastatic and lethal (Pourshams et al., 2019). It is urgent to understand the cause of pancreatic cancer progression and possible mechanisms. Most oral-gut microbiota forms a symbiotic relationship with humans after long-term mutual adaptation. More and more researchers have begun to pay attention to the link between human microbiota and pancreatic cancer (Zhang et al., 2020a), but the related mechanisms remain not well understood.

Lipopolysaccharide (LPS) is the main ingredient of bacterial cell walls and may increase pancreatic cancer risk by secreting pro-inflammatory cytokines (Yaw et al., 2020). Oral-gut pathogen infection has long-term effects to produce ROS or stimulate inflammatory cytokines, such as interleukin (IL)-1β, IL-6, IL-8, and tumor necrosis factor-α (TNF-α) (Supplementary Table 1). IL-1β induces ROS and nitric oxide (NO) production *via* phosphatidylinositol-3-kinase (PI3K)/Akt signaling (Rao et al., 2017). IL-6 promotes Epithelial-Mesenchymal transition (EMT) and metastasis through ROS production in cancer cells (Dong et al., 2018). TNF-α induces ROS production in human leukemia U937 cells (Moon et al., 2010). ROS often causes DNA damage, which is closely associated with radiotherapy resistance to pancreatic cancer therapy (Zhang et al., 2022). Furthermore, ROS has been confirmed to activate miR-21 (multiple-faceted biomarkers in various types of cancers) expression and functions (Zhang et al., 2020b). Phosphatase and tensin homolog deleted on chromosome ten (PTEN) is a direct target of miR-21 and the downregulation of miR-21 will upregulate PTEN levels and inhibit pancreatic cancer metastasis (Zhang et al., 2019).

Cancer cells with PTEN downregulation are often in the immunosuppressive tumor microenvironment regulated by myeloid-derived suppressor cells (MDSCs), regulatory T cells (Tregs), and M2 macrophages (Vidotto et al., 2020). Therefore, the miR-21/PTEN axis plays an important role in the immune escape of pancreatic cancer. The importance of the relationship between gut pathogens and pancreatic cancer risk was explored by investigating the miR-21/PTEN axis.

## The Gut Pathogens Associated with Pancreatic Cancer Risk

*Campylobacter jejuni* is a bacterial enteric pathogen often causing diarrhea and enterocolitis, and its Cas9 protein contributes to the carcinogenesis of pancreatic cancer (Chang et al., 2020). *Clostridium difficile* is a bacterium that causes mild to severe diarrhea and its infection is mostly found in patients with pancreatic ductal adenocarcinoma (PDAC) (Sumiyoshi et al., 2022). *Enterococcus faecalis* is a commensal Gram-positive pathogen and its infection may induce chronic pancreatitis and cancer (Maekawa et al., 2018). The presence of *Escherichia coli* in the bloodstream can promote the progression of pancreatic cancer (Bai et al., 2022). *Fusobacterium nucleatum* is a periodontal bacterium linked with a wide spectrum of human diseases and its infection occurrence in pancreatic cancer can cause shorter survival of patients (Mitsuhashi et al., 2015). *Helicobacter pylori* is a widely reported bacillus that can damage the tissue in the human stomach and duodenum, and its infection is linked to the development of pancreatic cancer (Kunovsky et al., 2021). *Porphyromonas gingivalis* is a widely reported dental pathogen, and pancreatic cancer development is promoted by its infection (Chen et al., 2020). Vibrio cholerae is a halophilic, facultative, and anaerobic bacillus, and is linked to pancreatic cancer metastasis (Cavuoti et al., 2002). The oral-gut pathogens related to pancreatic cancer risk were chosen for exploring the possible molecular mechanisms for the effects of oral-gut microbiota on pancreatic cancer progression (Supplementary Table 1).

## Gut Pathogens Possibly Promote Pancreatic Cancer to Escape from Immune Surveillance by Activating the MIR-21/PTEN Axis

The levels of miR-21 were found to be upregulated and the levels of PTEN were downregulated in the pancreatic cancer cell lines, such as CAPAN-1, BxPC-3, JF305, PANC-1, and SW1990, when compared with those in normal human pancreatic HPDE6-C7 cells. The upregulation of miR-21 and the downregulation of PTEN will promote these pancreatic cancer cell metastasis (Zhang et al., 2019). The miR-21 stimulates chemoresistance toward 5-fluorouracil in pancreatic cancer cells PATU8988 and PANC-1 by downregulating the levels of PTEN. The inhibition of miR-21 will rescue PTEN levels and increase the chemotherapy sensitivity of these pancreatic cancer cells (Wei et al., 2016). Similar results were also reported for other chemotherapy resistance in human pancreatic cancer cell lines MIAPaCa-E, MIAPaCa-M, and BxPC-3, which were resistant to gemcitabine. The downregulation of miR-21 caused the increase in tumor suppressor PTEN levels, which improved the sensitivity of these pancreatic cancer cells to gemcitabine (Ali et al., 2010). Among all the pathogens, *Bacteroidetes, E. coli, F. nucleatum, Helicobacter pylori, P. gingivalis, and P. aeruginosa* infection will result in an increase in miR-21 level and a decrease in PTEN levels (Supplementary Table 1). According to the above results, the upregulation of miR-21 and downregulation of PTEN will certainly promote pancreatic cancer cell metastasis and chemotherapy resistance. *Campylobacter jejuni, Citrobacter rodentium, Clostridium difficile, Enterococcus faecalis, Streptococcus B*, and *Staphylococcus aureus* infection can cause an increase in miR-21 levels, which will also result in the reduction in the level of PTEN (Supplementary Table 1). In this case, the pathogens' infection will cause pancreatic cancer cell metastasis, as well as chemoresistance. *Salmonella typhimurium* and *Treponema* infection will lead to a reduction in PTEN levels (Supplementary Table 1), which will promote pancreatic cancer cell proliferation since PTEN is a widely reported tumor suppressor.

Just as was previously mentioned, PTEN loss will increase immune suppressive cells, such as MDSCs, Tregs, and M2 macrophages. Pancreatic cancer cells are mediated by multiple immune cells under a powerful immunosuppressive environment, in which the cancer cells still can easily proliferate. MDSCs are significantly increased in both the circulation and the microenvironment of pancreatic cancer cells and are closely associated with the clinical cancer stages of PDAC (Markowitz et al., 2015). The prevalence of Tregs is also prevalent in the peripheral blood, as well as in the tumor microenvironment of pancreatic cancer, which is linked with pancreatic cancer progression and premalignant lesions (Lytle et al., 2019). M2 macrophages create an anti-inflammatory microenvironment of pancreatic cancer cells and also facilitate its progression (Attri et al., 2018). The present summary shows that the changes in the cell density of MDSCs, Tregs, and M2 macrophages have been found in the most pathogen infection, except for *Campylobacter jejuni* (Supplementary Table 1), suggesting that the most oral-gut pathogens may help pancreatic cancer escape from host immune by affecting immune suppressive cells *via* miR-21 and PTEN axis.

**Figure 1:**
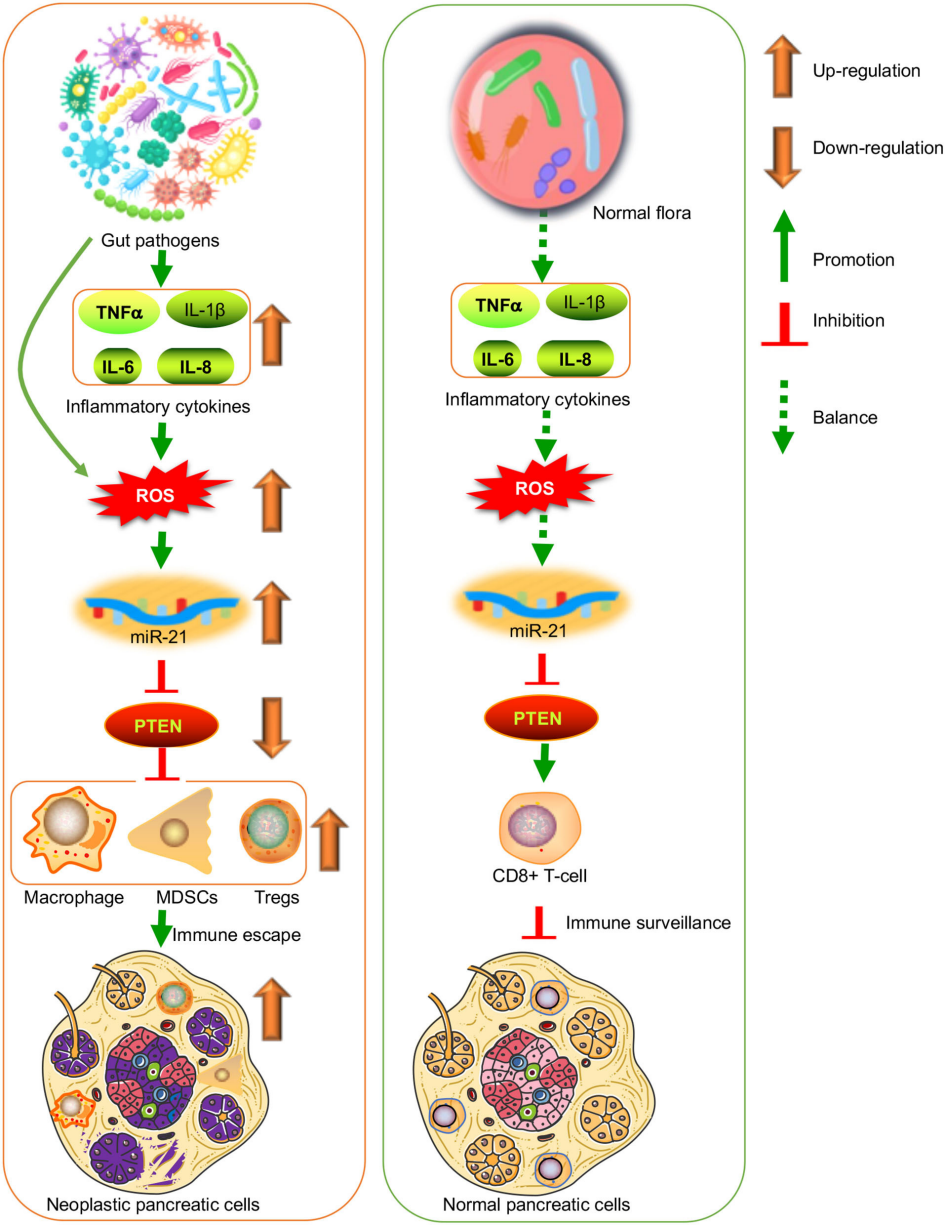
Oral-gut pathogens induce the immune escape of pancreatic cancer by affecting miR-21/PTEN axis *via* inflammatory cytokines-induced reactive oxygen species (ROS). It is suggestive that pathogen infection has long-term effects to stimulate inflammatory cytokines. Most of these cytokines can induced ROS products, which induce miR-21 expression and functions. Phosphatase and tensin homolog deleted on chromosome ten (PTEN) is a direct target of miR-21 and the upregulation of miR-21 will cause PTEN loss. Pancreatic cancer cells with PTEN downregulation are often in the immunosuppressive tumor microenvironment regulated by myeloid-derived suppressor cells (MDSCs), regulatory T cells (Tregs), and M2 macrophages. On the other hand, the healthy gut microbiota will maintain normal cancer immune surveillance possibly *via* CD8^+^ T cells.

From these results, a miR-21/PTEN axis may be involved with the immune escape of pancreatic cancer caused by an oral-gut pathogen. The upregulation of miR-21 inhibits PTEN expression, and its loss will increase in immune-suppressive cells, MDSCs, Tregs, and M2 macrophages. In this case, pancreatic cancer can evade host immune inhibition (Figure 1). For normal flora, if inflammatory cytokines and ROS maintain at a balanced level, the miR-21/PTEN axis will be inactivated. PTEN reaches a suitable level to control the immune-suppressive cells. CD8+ T cells, acting as mediators of adaptive immunity, selectively detect and eradicate cancer cells (Figure 1) (Philip and Schietinger, 2021). Furthermore, most pathogens' infections can cause an increase in inflammatory cytokine production. In the cytokine-rich microenvironment, pancreatic cancer develops a tolerance state to escape from host immune surveillance as well (Wang and Yao, 2019).

## Discussion

This present exploration indicates that oral-gut pathogens are closely associated with pancreatic cancer metastasis, possibly by arousing the immune escape of cancer cells *via* the miR-21/PTEN axis and immune-suppressive cells. The pattern of the effects of gut pathogens on the miR-21/PTEN axis will lead to better insights into the specific mechanisms associated with the immune escape of pancreatic cancer caused by oral-gut microbiota. Notably, not all pathogens will promote pancreatic cancer progression or metastasis. In contrast, some pathogens are beneficial to control pancreatic cancer development. *Listeria monocytogenes* expressing mesothelin are potential vaccines for preventing pancreatic cancer metastasis (Le et al., 2015). A genetically-modified *Salmonella typhimurium* may provide an effective approach to the prevention of orthotopic human pancreatic cancer (Nagakura et al., 2009). *Shigella flexneri* shows anti-tumor impacts on pancreatic cancer cells (Khodavirdipour et al., 2021). Therefore, some gut pathogens may provide effective tools to develop clinical immunotherapies for pancreatic cancer.

There are some limitations to this mini-review. Only a few types of oral-gut pathogens associated with the metastasis of pancreatic cancer were analyzed in this mini-review. We only analyzed that miR-21/PTEN axis regulates the immune escape of pancreatic cancer. PTEN can be regulated by other miRNA and another miRNA can mediate PTEN levels, which were not explored in this mini-review. Still, there is no complete job to approve that oral or gut pathogen causes the immune escape of pancreatic cancer *via* the miR-21/PTEN axis, although the clues between the pathogen infection and miR-21/PTEN axis activation can be traced from different kinds of literature. Much work needs to be done to better understand the specific mechanism for the effects of oral-gut microbiota on pancreatic cancer progression.

## Prospects and Challenges

A high prevalence of chemo-resistant pancreatic cancer may be caused by oral-gut pathogens. It is critical to diagnose and treat most people with oral-gut pathogens at the primary healthcare level. Most pathogens can also develop drug resistance and their therapy and control will become great challenges. Based on these common pathogen antigens, it may be an effective approach to explore multi-pathogen vaccines. According to the present summary, it is vital to maintain inflammatory and oxidative stress balance to prevent the development of pancreatic cancer. Furthermore, it will be a potential method to explore miR-21 and PTEN regulation *via* modern gene-editing techniques in a safe manner.

## Author Contributions

All authors listed have made a substantial, direct, and intellectual contribution to the work and approved it for publication.

## Conflict of Interest

The authors declare that the research was conducted in the absence of any commercial or financial relationships that could be construed as a potential conflict of interest.

## Publisher's Note

All claims expressed in this article are solely those of the authors and do not necessarily represent those of their affiliated organizations, or those of the publisher, the editors and the reviewers. Any product that may be evaluated in this article, or claim that may be made by its manufacturer, is not guaranteed or endorsed by the publisher.
