# Supplementary material for: An Exploration of Oral-Gut Pathogens Mediating Immune Escape of Pancreatic Cancer via miR-21/PTEN Axis
Source: Front Microbiol. 2022 Jun 22;13:928846. doi: 10.3389/fmicb.2022.928846 (PMC9258743; doi:10.3389/fmicb.2022.928846)
Supplement: Supplementary file 1 [file Table_1.doc]

Table S1, the effects of oral-gut pathogens on miR-21/PTEN axis and immune suppressive cells

| **Gut pathogens** | **PDI** | **miR-21** | **PTEN** | **ASIC** | **References** |
| --- | --- | --- | --- | --- | --- |
| Bacteroidetes | ROS, IL-6 and TNF-α | + | - | M2 macrophage | (Vanhooren et al., 2013;Wang et al., 2018a;Ding et al., 2020;Duan et al., 2020;Wei et al., 2020b;Hu et al., 2021)/(Azpiroz et al., 2021;McDew-White et al., 2022)/(Mitchell et al., 2018)/(Cao et al., 2020)/(Matsumoto et al., 2006;Panebianco et al., 2018;Genton et al., 2021;Kim et al., 2021;Lee et al., 2021) |
| *Campylobacter jejuni* | ROS, IL-6, IL-8 and TNF-α | + |  |  | (Korneev et al., 2018;Tawfeeq and Nasir, 2018;Hong et al., 2022)/(Rashid et al., 2021)///(Chang et al., 2020b;Shionoya et al., 2021) |
| *Citrobacter rodentium* | ROS, IL-1β, IL-6, IL-8 and TNF-α | + |  | Tregs | (Khan et al., 2008;Brown et al., 2011;Jain et al., 2015;Bording-Jorgensen et al., 2017)/(Wen et al., 2015;Yu et al., 2017)//(Ryz et al., 2012;Heller et al., 2014;Wang et al., 2014c;Mohinta et al., 2015;Knaus et al., 2017;Cho et al., 2019;Leber et al., 2019;Lin et al., 2019;Poysti et al., 2021;Zhang et al., 2021b;Gaignage et al., 2022)/(Hodgkinson, 2010) |
| *Clostridium difficile* | ROS, IL-1β, IL-6, IL-8 and TNF-α | + |  | M2 macrophage | (Linevsky et al., 1997;Jefferson et al., 1999;Warny et al., 2000;Carneiro-Filho et al., 2001;He et al., 2002;Hippenstiel et al., 2002;Grassl et al., 2003;Monick et al., 2003;Ng et al., 2003;Radeff et al., 2004;Woo et al., 2005;Sun et al., 2009;Absah and Faubion, 2012;Guo et al., 2012;Hansen et al., 2013;Boonma et al., 2014;Altwegg et al., 2018;Czepiel et al., 2018;Xu et al., 2018;Engevik et al., 2020;Foschetti et al., 2020;Gholam-Mostafaei et al., 2020;Htwe et al., 2021)//(Tsai et al., 2022)//(Saade Lemus et al., 2019;Uchiyama et al., 2021;Sumiyoshi et al., 2022) |
| *Enterococcus faecalis* | ROS, GM-CSF, IL-1β, IL-4, IL-6, IL-8, IL-12, TNF-α | + |  | Tregs, M2 macrophage | (Kern et al., 1993;Okutomi et al., 1997;Yui et al., 2001;Kim et al., 2006b;Yang et al., 2012;Strickertsson et al., 2013;Tsuruta et al., 2013;Im et al., 2015;Wang et al., 2015;Altwegg et al., 2018;Léger et al., 2019;Lu et al., 2019;Hyun et al., 2021;Kabwe et al., 2021;Flores-Maldonado et al., 2022)/(Lv et al., 2017;Li et al., 2020a)//(Polak et al., 2021;Kiyoi et al., 2022)/(Rolston et al., 1995;Halimi et al., 2021;Shrader et al., 2021) |
| *Escherichia coli* | ROS, TNF-α, IL-1β, IL-6, IL-8 | + | - | MDSCs, M2 macrophage | (Harada et al., 1993;Hedges et al., 1996;Godaly et al., 1997;Ruiz-Laguna et al., 2000;Steiner et al., 2000;Brauner et al., 2001;Galanakis et al., 2006;Meraz et al., 2007;Zaga-Clavellina et al., 2007;Berrisford et al., 2008;Diard et al., 2009;Semiramoth et al., 2009;Khan et al., 2010;Knuuti et al., 2013;Tanimoto et al., 2013;Leung et al., 2014;Li et al., 2014;Chen et al., 2015b;Elatrech et al., 2015;Karlsson et al., 2015;Reynolds et al., 2017;Wan et al., 2017;Jin et al., 2018;Lee et al., 2018b;Chang et al., 2019b;Govindan et al., 2019;Yusuf et al., 2019)/(Rashid et al., 2021)/(Schulz et al., 2021)/(Long et al., 2021),(van Harten et al., 2022)/(Gregg, 1977;Rolston et al., 1995;Yan et al., 1995;Evoy et al., 1997;Giantonio et al., 1997;Green et al., 1997;Bruell et al., 2005;Deharvengt et al., 2005;He et al., 2006;Zhou et al., 2007;Kaliberova et al., 2008;Wang et al., 2008;Wang et al., 2010;Chen et al., 2014;Liu et al., 2018;Osawa, 2018;Panebianco et al., 2018;Belbekhouche et al., 2020;Luo et al., 2020;Bai et al., 2022) |
| *Fusobacterium nucleatum* | ROS, IL-1β, IL-6, IL-8, TNF-α | + | - | MDSCs, Tregs, M2 macrophages | (Yamazaki et al., 1989;Yoshimura et al., 1997;Socransky et al., 2000;Yamaguchi et al., 2005;Borch et al., 2010;Tang et al., 2016;Sampaio Fernandes et al., 2017;Lesiow et al., 2018;Kang et al., 2019;Lesiow et al., 2019;Yusuf et al., 2019;Casasanta et al., 2020;Chang et al., 2020a;Figueiredo et al., 2020;Lesiow et al., 2020;Romero-Castro et al., 2020;Engevik et al., 2021)/(Sun et al., 2019)/(Liang et al., 2020)/(Chen et al., 2018;Sakamoto et al., 2021b;Zhang et al., 2021a)/(Gaiser et al., 2019;Sethi et al., 2019;Chung et al., 2021) |
| *Helicobacter pylori* | ROS, IL-1β, IL-6, IL-8, TNF-α | + | - | MDSCs, Tregs, M2 macrophages | (Wadstrom et al., 1994;Beales and Calam, 1997;Caccavo et al., 1997;Shibata et al., 1999;Amjad et al., 2001;Kraft et al., 2001;Yea et al., 2001;Hamajima et al., 2003;Kuzuhara et al., 2005;Li et al., 2005;Lu et al., 2005;Suganuma and Fujiki, 2005;Atsuta et al., 2006;Hirata et al., 2006;Ishida et al., 2006;Kim et al., 2006a;Kuzuhara et al., 2007;Hellmig et al., 2008;Kuo et al., 2008;Suganuma et al., 2008;Jang et al., 2009;Melo Barbosa et al., 2009;Tsuge et al., 2009;Xiao et al., 2009;Zalewska-Ziob et al., 2009;Ahmadzadeh et al., 2010;Collodel et al., 2010;Handa et al., 2010;Abdollahi et al., 2011;Kumar Pachathundikandi et al., 2011;Gao et al., 2012;Santos et al., 2012;Suganuma et al., 2012;Tahara et al., 2012;Ianovich et al., 2013;Pathak et al., 2013;Zhao et al., 2013;Lin et al., 2014;Triantafillidis et al., 2014;Watanabe et al., 2014;Kumar Pachathundikandi et al., 2015;Li et al., 2015;Siregar et al., 2015;Staff, 2015;Zabaglia et al., 2015;Ngo et al., 2016;Sulzbach et al., 2016;Chen et al., 2017;Gobert and Wilson, 2017;Tavares and Pathak, 2017;Kim et al., 2018;Moradipour et al., 2018;Tavares and Pathak, 2018b;Tavares and Pathak, 2018a;Tourani et al., 2018;Wen et al., 2018a;Xie et al., 2018;Zabaglia et al., 2018;Bounder et al., 2020;Lu et al., 2020;Al-Eitan et al., 2021;Lin et al., 2021;Negovan et al., 2021;Suganuma et al., 2021;Bi et al., 2022;Morningstar-Wright et al., 2022;Xia et al., 2022)/(Belair et al., 2009)//(Lu et al., 2021;Urakawa et al., 2021;Yang et al., 2021)/(Raderer et al., 1998;Stolzenberg-Solomon et al., 2001;Lowenfels and Maisonneuve, 2002;Nilsson et al., 2002;Risch, 2003;Stolzenberg-Solomon et al., 2003;Wohrer et al., 2003;Zalatnai, 2003;Capurso et al., 2004;Luo et al., 2007;Takayama et al., 2007;de Martel et al., 2008;Diamantidis et al., 2008;Hart et al., 2008;Lindkvist et al., 2008;Johansen et al., 2010;Maisonneuve and Lowenfels, 2010;Risch et al., 2010;Trikudanathan et al., 2011;Gawin et al., 2012;Li et al., 2012;Risch, 2012;Yeo and Lowenfels, 2012;Felix et al., 2013;Michaud, 2013;Risch et al., 2013;Xiao et al., 2013;Yu et al., 2013;Wang et al., 2014b;Ai et al., 2015;Chen et al., 2015a;Maisonneuve and Lowenfels, 2015;Schulte et al., 2015;Wang and Li, 2015;Chen et al., 2016;Guo et al., 2016;Ertz-Archambault et al., 2017;Gomez-Rubio et al., 2017;Huang et al., 2017;Liu et al., 2017;Risch et al., 2017;Archibugi et al., 2018;Mei et al., 2018;Yamanoi and Nakayama, 2018;Hirabayashi et al., 2019;Karpinski, 2019;Panebianco and Pazienza, 2019;Rawla et al., 2019;Sethi et al., 2019;Bezmin Abadi, 2020;Brusselaers et al., 2020;Hirabayashi et al., 2020;Kumar et al., 2020;Li et al., 2020b;Morgell et al., 2020;Gong et al., 2021;Kunovsky et al., 2021;Morgell et al., 2021;Permuth et al., 2021;Stasiewicz et al., 2021;Laya et al., 2022) |
| *Porphyromonas gingivalis* | ROS, IL-1β, IL-6, IL-8 and TNF-α | + | - | Tregs, M2 macrophages | (Murakami et al., 1993;Aoyagi et al., 1995;Ogawa and Uchida, 1996;Shapira et al., 1997;Banbula et al., 1999;Kent et al., 1999;Wang et al., 1999;Hirai et al., 2000;Chen and Yan, 2001;Petelin et al., 2004;Yamaguchi et al., 2005;Diya et al., 2008;Baer et al., 2009;Bailey et al., 2009;Le et al., 2009;Tamai et al., 2009;Borch et al., 2010;Inomata et al., 2010;Morandini et al., 2010;Herath et al., 2011;Kukita et al., 2012;Luo et al., 2012;Nebel et al., 2013;Trindade et al., 2013;Chi et al., 2014;Kato et al., 2014;Liu et al., 2014;Wang et al., 2014a;Yee et al., 2014;Zhang et al., 2014;Yiemwattana and Kaomongkolgit, 2015;Bugueno et al., 2016;Rapado-González et al., 2016;Dekita et al., 2017;Glowczyk et al., 2017;Rokad et al., 2017;Wang et al., 2017b;Lian et al., 2018;Bachtiar et al., 2020;Takahashi et al., 2022)/(Chang et al., 2019a)/(Chang et al., 2019a)/(Holden et al., 2014)/(Michaud, 2013;Michaud et al., 2013;Ogrendik, 2015;2016;Jacob, 2016;Ogrendik, 2017;Choy et al., 2018;Fan et al., 2018;Mohammed et al., 2018;Karpinski, 2019;Chen et al., 2020;Hiraki et al., 2020;Stasiewicz et al., 2021;Petrick et al., 2022) |
| *Pseudomonas aeruginosa* | ROS, IL-1β, IL-6, IL-8 and TNF-α | + | - | Tregs, M2 macrophages | (Staugas et al., 1992;Shirai et al., 1997;Sonoda et al., 1997;Hickling et al., 1998;Nishimura et al., 1998;Schumann et al., 1998;Cole et al., 1999;Skerrett et al., 1999;Harder et al., 2000;Schumann et al., 2000;Leidal et al., 2001;Xue et al., 2001a;Xue et al., 2001b;Azghani et al., 2002;Raga et al., 2003;Joseph et al., 2005;Sun et al., 2005;Bauman and Kuehn, 2006;Tseng et al., 2006;Lagoumintzis et al., 2008;Zhu et al., 2008;Gerstel et al., 2009;Venza et al., 2009;Gambari et al., 2010;Choi et al., 2011;Jouneau et al., 2011;Reinis et al., 2011;de Lima et al., 2012;Rupesh et al., 2012;Tsay et al., 2013;Colomb et al., 2014;Fan et al., 2014;Chekabab et al., 2015;Khan et al., 2015;Roussel et al., 2016;Yang et al., 2017;Chakraborty et al., 2018;Lee et al., 2018a;Li et al., 2018;Rimessi et al., 2018;Yadav et al., 2018;Hayakawa et al., 2019;Nakamoto et al., 2019;Han et al., 2020;Huang et al., 2020;Lee et al., 2020;Liu et al., 2020;Yang et al., 2020;Chatterjee et al., 2021;Ghasemi et al., 2021)/(Riquelme et al., 2019)/(Hubbard et al., 2011)/(Wang et al., 2016)/(Kornmann et al., 1999;Bruell et al., 2005;Shimamura et al., 2010;Tajima et al., 2013;Hollevoet et al., 2015;Cheng et al., 2016;Gaida et al., 2016;Moayedi et al., 2018) |
| *Salmonella typhimurium* | ROS, IL-1β, IL-6, IL-8 and TNF-α |  | - | Tregs | (Mitsui-Yamaguchi et al., 1997;Xu et al., 1998;Galdiero et al., 2001;Merlin et al., 2001;Janssen et al., 2002;Romanova et al., 2002;Cho and Chae, 2003;van Diepen et al., 2007;Romanova Iu et al., 2008;Leschner et al., 2009;dos Santos et al., 2011;Yoon et al., 2011;Morales et al., 2013;Yoon et al., 2014;Joseph et al., 2016;Lopez-Colom et al., 2019;Noster et al., 2019;Jeffrey et al., 2020)//(Howe et al., 2019)/(Liu and Chopra, 2010;Nakahashi-Oda et al., 2016)/(Nagakura et al., 2009;Yam et al., 2010;Hiroshima et al., 2013;Hiroshima et al., 2014a;Hiroshima et al., 2014b;Hoffman and Bouvet, 2015;Kawaguchi et al., 2018;Murakami et al., 2018;Ebelt et al., 2021;Tan et al., 2022) |
| Streptococcus B | ROS, IL-1β, IL-6, IL-8 and TNF-α | + |  | M2 macrophage | (Vallejo et al., 2000;Maisey et al., 2008;Nakstad et al., 2016;Castro-Leyva et al., 2022)/(Castro-Leyva et al., 2022)//(Flaherty et al., 2019;Flaherty et al., 2021)/(Wei et al., 2020a) |
| *Staphylococcus aureus* | ROS, IL-1β, IL-6, IL-8 and TNF-α | + |  | Tregs, M2 macrophages | (Tufano et al., 1991;Hinshaw et al., 1992;Ferrante et al., 1994;Matuschak et al., 1994;Kimura et al., 1997;Hultgren et al., 1998;Haziot et al., 1999;Tikhonov et al., 2001;Kielian et al., 2004;O'Brien et al., 2006;Liang and Ji, 2007;Satorres et al., 2007;Oviedo-Boyso et al., 2008a;Oviedo-Boyso et al., 2008b;Li et al., 2010;Shibata et al., 2010;Kapetanovic et al., 2011;Wu et al., 2012;Zhang et al., 2013;Varley et al., 2014;Kang et al., 2015;Nymo et al., 2016;Svedova et al., 2016;Nandi and Bishayi, 2017;Wang et al., 2018b;Wen et al., 2018b;Sultana and Bishayi, 2020;Dutta and Bishayi, 2021;Singh et al., 2021;Verma et al., 2021;Liu et al., 2022;Xu et al., 2022)/(Jin et al., 2014)//(Dey and Bishayi, 2017;2020b;a;Sakamoto et al., 2021a;Asila et al., 2022)/(Li et al., 2008;Li et al., 2009;Kinoshita et al., 2012;Liquete et al., 2012;Wang et al., 2019;Belbekhouche et al., 2020) |
| *Treponema* | ROS, IL-1β, IL-6, IL-8 and TNF-α |  | - | M2 macrophages | (Miyamoto et al., 2006;Tamai et al., 2009;Jo et al., 2014;Pozzobon et al., 2016;Xie et al., 2017;Luo et al., 2018;Pozzobon et al., 2018;Li et al., 2021;Lu et al., 2022)//(Jones et al., 2019)/(Akins et al., 1993;Xu et al., 2019)/(Ogrendik, 2015;2017) |
| *Vibrio cholerae* | ROS, TNF-α, IL-1β, IL-6, IL-8, and TGF-β |  |  | MDSCs, Tregs | (Ogura et al., 2017;Wang et al., 2017a;Yang et al., 2018;Bagheri-Josheghani and Bakhshi, 2022)///(King et al., 2019),(Prawiro et al., 2020)/(Cavuoti et al., 2002;Akasov et al., 2016) |

Note: The symbol ‘+’ stands for upregulation and ‘-’ for downregulation. Pathogen-derived inducers, PDI. The references are shown in **A/B/C/D/E** order, **A** stands for the references associated with pathogen-derided inducers, **B** for the references associated with the effects of pathogen infection on miR-21, **C** for the references associated with the effects of pathogen infection on Phosphatase and tensin homolog (PTEN), **D** for the references associated with the effects of pathogen infection on suppressive immune cells (ASIC), which include myeloid-derived suppressor cells (MDSCs), T regulatory (Treg) cells and M2 macrophages.

**References**

(2016). P. Gingivalis May Increase Pancreatic Cancer Risk. *J Calif Dent Assoc* 44**,** 324.

Abdollahi, H., Shams, S., Zahedi, M.J., Darvish Moghadam, S., Hayatbakhsh, M.M., and Jafarzadeh, A. (2011). IL-10, TNF-alpha and IFN-gamma levels in serum and stomach mucosa of Helicobacter pylori-infected patients. *Iran J Allergy Asthma Immunol* 10**,** 267-271.

Absah, I., and Faubion, W.A., Jr. (2012). Concomitant therapy with methotrexate and anti-TNF-alpha in pediatric patients with refractory crohn's colitis: a case series. *Inflamm Bowel Dis* 18**,** 1488-1492.

Ahmadzadeh, E., Zarkesh-Esfahani, H., Roghanian, R., and Akbar, F.N. (2010). Comparison of Helicobacter pylori and Escherichia coli in induction of TNF-alpha mRNA from human peripheral blood mononuclear cells. *Indian J Med Microbiol* 28**,** 233-237.

Ai, F., Hua, X., Liu, Y., Lin, J., and Feng, Z. (2015). Preliminary study of pancreatic cancer associated with Helicobacter pylori infection. *Cell Biochem Biophys* 71**,** 397-400.

Akasov, R., Haq, S., Haxho, F., Samuel, V., Burov, S.V., Markvicheva, E., Neufeld, R.J., and Szewczuk, M.R. (2016). Sialylation transmogrifies human breast and pancreatic cancer cells into 3D multicellular tumor spheroids using cyclic RGD-peptide induced self-assembly. *Oncotarget* 7**,** 66119-66134.

Akins, D.R., Purcell, B.K., Mitra, M.M., Norgard, M.V., and Radolf, J.D. (1993). Lipid modification of the 17-kilodalton membrane immunogen of Treponema pallidum determines macrophage activation as well as amphiphilicity. *Infect Immun* 61**,** 1202-1210.

Al-Eitan, L.N., Almomani, F.A., and Al-Khatib, S.M. (2021). Association of CYP2C19, TNF-alpha, NOD1, NOD2, and PPARgamma polymorphisms with peptic ulcer disease enhanced by Helicobacter pylori infection. *Saudi Med J* 42**,** 21-29.

Altwegg, R., Combes, R., Laharie, D., De Ledinghen, V., Radenne, S., Conti, F., Chazouilleres, O., Duvoux, C., Dumortier, J., Leroy, V., Treton, X., Durand, F., Dharancy, S., Nachury, M., Goutorbe, F., Lamblin, G., Boivineau, L., Peyrin-Biroulet, L., and Pageaux, G.P. (2018). Effectiveness and safety of anti-TNF therapy for inflammatory bowel disease in liver transplant recipients for primary sclerosing cholangitis: A nationwide case series. *Dig Liver Dis* 50**,** 668-674.

Amjad, M., Kazmi, S.U., Qureshi, S.M., and Reza-Ul Karim, M. (2001). Inhibitory effect of IL-4 on the production of IL-1 beta and TNF-alpha by gastric mononuclear cells of Helicobacter pylori infected patients. *Ir J Med Sci* 170**,** 112-116.

Aoyagi, T., Sugawara-Aoyagi, M., Yamazaki, K., and Hara, K. (1995). Interleukin 4 (IL-4) and IL-6-producing memory T-cells in peripheral blood and gingival tissue in periodontitis patients with high serum antibody titers to Porphyromonas gingivalis. *Oral Microbiol Immunol* 10**,** 304-310.

Archibugi, L., Signoretti, M., and Capurso, G. (2018). The Microbiome and Pancreatic Cancer: An Evidence-based Association? *J Clin Gastroenterol* 52 Suppl 1, Proceedings from the 9th Probiotics, Prebiotics and New Foods, Nutraceuticals and Botanicals for Nutrition & Human and Microbiota Health Meeting, held in Rome, Italy from September 10 to 12, 2017**,** S82-S85.

Asila, A., Liu, J., Liu, J., Li, L., and Liao, J. (2022). Immunomodulatory effects of berberine on Staphylococcus aureus-induced septic arthritis through down-regulation of Th17 and Treg signaling pathways. *Acta Biochim Pol* 69**,** 215-226.

Atsuta, Y., Ito, L.S., Oba-Shinjo, S.M., Uno, M., Shinjo, S.K., Marie, S.K., Goto, Y., and Hamajima, N. (2006). Associations of TNF-A-1031TT and -857TT genotypes with Helicobacter pylori seropositivity and gastric atrophy among Japanese Brazilians. *Int J Clin Oncol* 11**,** 140-145.

Azghani, A.O., Baker, J.W., Shetty, S., Miller, E.J., and Bhat, G.J. (2002). Pseudomonas aeruginosa elastase stimulates ERK signaling pathway and enhances IL-8 production by alveolar epithelial cells in culture. *Inflamm Res* 51**,** 506-510.

Azpiroz, M.A., Orguilia, L., Palacio, M.I., Malpartida, A., Mayol, S., Mor, G., and Gutierrez, G. (2021). Potential biomarkers of infertility associated with microbiome imbalances. *Am J Reprod Immunol* 86**,** e13438.

Bachtiar, E.W., Putri, C.F., Soejoedono, R.D., and Bachtiar, B.M. (2020). Expression of TNF, IL1B, and iNOS2 in the neural cell after induced by Porphyromonas gingivalis with and without coating antibody anti -Porphyromonas gingivalis. *F1000Res* 9**,** 1499.

Baer, M.T., Huang, N., and Gibson, F.C., 3rd (2009). Scavenger receptor A is expressed by macrophages in response to Porphyromonas gingivalis, and participates in TNF-alpha expression. *Oral Microbiol Immunol* 24**,** 456-463.

Bagheri-Josheghani, S., and Bakhshi, B. (2022). Formulation of selenium nanoparticles encapsulated by alginate-chitosan for controlled delivery of Vibrio Cholerae LPS: A novel delivery system candidate for nanovaccine. *International Journal of Biological Macromolecules*.

Bai, C., Zhang, X., Yang, D., Li, D., Feng, H., and Li, Y. (2022). Clinical Analysis of Bloodstream Infection of Escherichia coli in Patients with Pancreatic Cancer from 2011 to 2019. *Can J Infect Dis Med Microbiol* 2022**,** 1338188.

Bailey, M.T., Kinsey, S.G., Padgett, D.A., Sheridan, J.F., and Leblebicioglu, B. (2009). Social stress enhances IL-1beta and TNF-alpha production by Porphyromonas gingivalis lipopolysaccharide-stimulated CD11b+ cells. *Physiol Behav* 98**,** 351-358.

Banbula, A., Bugno, M., Kuster, A., Heinrich, P.C., Travis, J., and Potempa, J. (1999). Rapid and efficient inactivation of IL-6 gingipains, lysine- and arginine-specific proteinases from Porphyromonas gingivalis. *Biochem Biophys Res Commun* 261**,** 598-602.

Bauman, S.J., and Kuehn, M.J. (2006). Purification of outer membrane vesicles from Pseudomonas aeruginosa and their activation of an IL-8 response. *Microbes Infect* 8**,** 2400-2408.

Beales, I.L., and Calam, J. (1997). Stimulation of IL-8 production in human gastric epithelial cells by Helicobacter pylori, IL-1beta and TNF-alpha requires tyrosine kinase activity, but not protein kinase C. *Cytokine* 9**,** 514-520.

Belair, C., Darfeuille, F., and Staedel, C. (2009). Helicobacter pylori and gastric cancer: possible role of microRNAs in this intimate relationship. *Clinical microbiology and infection* 15**,** 806-812.

Belbekhouche, S., Poostforooshan, J., Shaban, M., Ferrara, B., Alphonse, V., Cascone, I., Bousserrhine, N., Courty, J., and Weber, A.P. (2020). Fabrication of large pore mesoporous silica microspheres by salt-assisted spray-drying method for enhanced antibacterial activity and pancreatic cancer treatment. *Int J Pharm* 590**,** 119930.

Berrisford, J.M., Thompson, C.J., and Sazanov, L.A. (2008). Chemical and NADH-induced, ROS-dependent, cross-linking between subunits of complex I from Escherichia coli and Thermus thermophilus. *Biochemistry* 47**,** 10262-10270.

Bezmin Abadi, A.T. (2020). Helicobacter pylori infection and pancreatic cancer. *J Cancer Res Ther* 16**,** S253.

Bi, Y., Wu, D., Wu, X., Wang, F., Yu, H., Liu, P., Cui, G., and Chen, Z. (2022). Phycocyanin inhibits Helicobacter pylori-induced hyper-proliferation in AGS cells via activation of the ROS/MAPK signaling pathway. *Ann Transl Med* 10**,** 176.

Boonma, P., Spinler, J.K., Venable, S.F., Versalovic, J., and Tumwasorn, S. (2014). Lactobacillus rhamnosus L34 and Lactobacillus casei L39 suppress Clostridium difficile-induced IL-8 production by colonic epithelial cells. *BMC Microbiol* 14**,** 177.

Borch, T.S., Holmstrup, P., Bendtzen, K., and Nielsen, C.H. (2010). In vitro cytokine responses to periodontal pathogens: generalized aggressive periodontitis is associated with increased IL-6 response to Porphyromonas gingivalis. *Scand J Immunol* 71**,** 440-446.

Bording-Jorgensen, M., Alipour, M., Danesh, G., and Wine, E. (2017). Inflammasome activation by ATP enhances Citrobacter rodentium clearance through ROS generation. *Cellular Physiology and Biochemistry* 41**,** 193-204.

Bounder, G., Jouimyi, M.R., Boura, H., Touati, E., Michel, V., Badre, W., Jouhadi, H., Kadi, M., Eljihad, M., Benomar, H., Kettani, A., Lebrazi, H., and Maachi, F. (2020). Associations of the -238(G/A) and -308(G/A) TNF-alpha Promoter Polymorphisms and TNF-alpha Serum Levels with the Susceptibility to Gastric Precancerous Lesions and Gastric Cancer Related to Helicobacter pylori Infection in a Moroccan Population. *Asian Pac J Cancer Prev* 21**,** 1623-1629.

Brauner, A., Soderhall, M., Jacobson, S.H., Lundahl, J., Andersson, U., and Andersson, J. (2001). Escherichia coli-induced expression of IL-1 alpha, IL-1 beta, IL-6 and IL-8 in normal human renal tubular epithelial cells. *Clin Exp Immunol* 124**,** 423-428.

Brown, J.B., Cheresh, P., Goretsky, T., Managlia, E., Grimm, G.R., Ryu, H., Zadeh, M., Dirisina, R., and Barrett, T.A. (2011). Epithelial phosphatidylinositol-3-kinase signaling is required for β-catenin activation and host defense against Citrobacter rodentium infection. *Infection and immunity* 79**,** 1863-1872.

Bruell, D., Bruns, C.J., Yezhelyev, M., Huhn, M., Muller, J., Ischenko, I., Fischer, R., Finnern, R., Jauch, K.W., and Barth, S. (2005). Recombinant anti-EGFR immunotoxin 425(scFv)-ETA' demonstrates anti-tumor activity against disseminated human pancreatic cancer in nude mice. *Int J Mol Med* 15**,** 305-313.

Brusselaers, N., Sadr-Azodi, O., and Engstrand, L. (2020). Long-term proton pump inhibitor usage and the association with pancreatic cancer in Sweden. *J Gastroenterol* 55**,** 453-461.

Bugueno, I.M., Khelif, Y., Seelam, N., Morand, D.N., Tenenbaum, H., Davideau, J.L., and Huck, O. (2016). Porphyromonas gingivalis Differentially Modulates Cell Death Profile in Ox-LDL and TNF-alpha Pre-Treated Endothelial Cells. *PLoS One* 11**,** e0154590.

Caccavo, D., Leri, O., Ferri, G.M., Perinelli, P., De Luca, D., and Afeltra, A. (1997). Anti-TNF-alpha antibodies are not associated with Helicobacter pylori induced gastritis. *Eur Rev Med Pharmacol Sci* 1**,** 111-113.

Cao, X., Tang, L., Zeng, Z., Wang, B., Zhou, Y., Wang, Q., Zou, P., and Li, W. (2020). Effects of Probiotics BaSC06 on Intestinal Digestion and Absorption, Antioxidant Capacity, Microbiota Composition, and Macrophage Polarization in Pigs for Fattening. *Front Vet Sci* 7**,** 570593.

Capurso, G., Delle Fave, G., and Lemoine, N. (2004). Re: Etiology of pancreatic cancer, with a hypothesis concerning the role of N-nitroso compounds and excess gastric acidity. *J Natl Cancer Inst* 96**,** 75; author reply 75-76.

Carneiro-Filho, B.A., Souza, M.L., Lima, A.A., and Ribeiro, R.A. (2001). The effect of tumour necrosis factor (TNF) inhibitors in Clostridium difficile toxin-induced paw oedema and neutrophil migration. *Pharmacol Toxicol* 88**,** 313-318.

Casasanta, M.A., Yoo, C.C., Udayasuryan, B., Sanders, B.E., Umana, A., Zhang, Y., Peng, H., Duncan, A.J., Wang, Y., Li, L., Verbridge, S.S., and Slade, D.J. (2020). Fusobacterium nucleatum host-cell binding and invasion induces IL-8 and CXCL1 secretion that drives colorectal cancer cell migration. *Sci Signal* 13.

Castro-Leyva, V., Arenas-Huertero, F., Espejel-Nunez, A., Giono Cerezo, S., Flores-Pliego, A., Espino, Y.S.S., Reyes-Munoz, E., Vadillo-Ortega, F., Borboa-Olivares, H., Camacho-Arroyo, I., and Estrada-Gutierrez, G. (2022). miR-21 differentially regulates IL-1beta and IL-10 expression in human decidual cells infected with streptococcus B. *Reprod Biol* 22**,** 100604.

Cavuoti, D., Fogli, M., Quinton, R., Gander, R.M., and Southern, P.M. (2002). Splenic abscess with Vibrio cholerae masking pancreatic cancer. *Diagn Microbiol Infect Dis* 43**,** 311-313.

Chakraborty, P., Joardar, S., Ray, S., Biswas, P., Maiti, D., and Tribedi, P. (2018). 3,6-Di(pyridin-2-yl)-1,2,4,5-tetrazine (pytz)-capped silver nanoparticles (TzAgNPs) inhibit biofilm formation of Pseudomonas aeruginosa: a potential approach toward breaking the wall of biofilm through reactive oxygen species (ROS) generation. *Folia Microbiol (Praha)* 63**,** 763-772.

Chang, A.M., Bamashmous, S., Darveau, R.P., and Rajapakse, S. (2020a). An Ayurvedic herbal extract inhibits oral epithelial cell IL-8 responses to host and bacterial agonists. *BMC Complement Med Ther* 20**,** 62.

Chang, C., Wang, H., Liu, J., Pan, C., Zhang, D., Li, X., and Pan, Y. (2019a). Porphyromonas gingivalis infection promoted the proliferation of oral squamous cell carcinoma cells through the miR-21/PDCD4/AP-1 negative signaling pathway. *ACS Infectious Diseases* 5**,** 1336-1347.

Chang, T.-K., Cheng, T.-M., Chu, H.-L., Tan, S.-H., Kuo, J.-C., Hsu, P.-H., Su, C.-Y., Chen, H.-M., Lee, C.-M., and Kuo, T.-R. (2019b). Metabolic mechanism investigation of antibacterial active cysteine-conjugated gold nanoclusters in Escherichia coli. *ACS Sustainable Chemistry & Engineering* 7**,** 15479-15486.

Chang, Y.J., Bae, J., Zhao, Y., Lee, G., Han, J., Lee, Y.H., Koo, O.J., Seo, S., Choi, Y.K., and Yeom, S.C. (2020b). In vivo multiplex gene targeting with Streptococcus pyogens and Campylobacter jejuni Cas9 for pancreatic cancer modeling in wild-type animal. *J Vet Sci* 21**,** e26.

Chatterjee, S., Paul, P., Chakraborty, P., Das, S., Sarker, R.K., Sarkar, S., Das, A., and Tribedi, P. (2021). Cuminaldehyde exhibits potential antibiofilm activity against Pseudomonas aeruginosa involving reactive oxygen species (ROS) accumulation: a way forward towards sustainable biofilm management. *3 Biotech* 11**,** 485.

Chekabab, S.M., Silverman, R.J., Lafayette, S.L., Luo, Y., Rousseau, S., and Nguyen, D. (2015). Staphylococcus aureus Inhibits IL-8 Responses Induced by Pseudomonas aeruginosa in Airway Epithelial Cells. *PLoS One* 10**,** e0137753.

Chen, G., Tang, N., Wang, C., Xiao, L., Yu, M., Zhao, L., Cai, H., Han, L., Xie, C., and Zhang, Y. (2017). TNF-alpha-inducing protein of Helicobacter pylori induces epithelial-mesenchymal transition (EMT) in gastric cancer cells through activation of IL-6/STAT3 signaling pathway. *Biochem Biophys Res Commun* 484**,** 311-317.

Chen, L.L., and Yan, J. (2001). Porphyromonas gingivalis lipopolysaccharide activated bone resorption of osteoclasts by inducing IL-1, TNF, and PGE. *Acta Pharmacol Sin* 22**,** 614-618.

Chen, S.M., Hsu, L.J., Lee, H.L., Lin, C.P., Huang, S.W., Lai, C.J., Lin, C.W., Chen, W.T., Chen, Y.J., Lin, Y.C., Yang, C.C., and Jan, M.S. (2020). Lactobacillus Attenuate the Progression of Pancreatic Cancer Promoted by Porphyromonas Gingivalis in K-ras(G12D) Transgenic Mice. *Cancers (Basel)* 12.

Chen, T., Li, Q., Wu, J., Wu, Y., Peng, W., Li, H., Wang, J., Tang, X., Peng, Y., and Fu, X. (2018). Fusobacterium nucleatum promotes M2 polarization of macrophages in the microenvironment of colorectal tumours via a TLR4-dependent mechanism. *Cancer Immunology, Immunotherapy* 67**,** 1635-1646.

Chen, X.Z., Schottker, B., Castro, F.A., Chen, H., Zhang, Y., Holleczek, B., and Brenner, H. (2016). Association of helicobacter pylori infection and chronic atrophic gastritis with risk of colonic, pancreatic and gastric cancer: A ten-year follow-up of the ESTHER cohort study. *Oncotarget* 7**,** 17182-17193.

Chen, X.Z., Wang, R., Chen, H.N., and Hu, J.K. (2015a). Cytotoxin-Associated Gene A-Negative Strains of Helicobacter pylori as a Potential Risk Factor of Pancreatic Cancer: A Meta-Analysis Based on Nested Case-Control Studies. *Pancreas* 44**,** 1340-1344.

Chen, Y., Wang, X., Zhao, P., Zhang, Y., and Cao, B. (2014). Development and characterization of monoclonal antibodies against pancreatic cancer marker hippocalcin-like 1 protein. *Monoclon Antib Immunodiagn Immunother* 33**,** 20-27.

Chen, Z., Zhou, Q., Zou, D., Tian, Y., Liu, B., Zhang, Y., and Wu, Z. (2015b). Chloro-benzoquinones cause oxidative DNA damage through iron-mediated ROS production in Escherichia coli. *Chemosphere* 135**,** 379-386.

Cheng, X., Wang, B., Jin, Z., Ma, D., Yang, W., Zhao, R., Jing, X., Shen, B., Peng, C., and Qiu, W. (2016). Pseudomonas aeruginosa-mannose-sensitive hemagglutinin inhibits pancreatic cancer cell proliferation and induces apoptosis via the EGFR pathway and caspase signaling. *Oncotarget* 7**,** 77916-77925.

Chi, X.P., Ouyang, X.Y., and Wang, Y.X. (2014). Hydrogen sulfide synergistically upregulates Porphyromonas gingivalis lipopolysaccharide-induced expression of IL-6 and IL-8 via NF-kappaB signalling in periodontal fibroblasts. *Arch Oral Biol* 59**,** 954-961.

Cho, H., Jaime, H., De Oliveira, R.P., Kang, B., Spolski, R., Vaziri, T., Myers, T.G., Thovarai, V., Shen, Z., Fox, J.G., Leonard, W.J., and Kelsall, B.L. (2019). Defective IgA response to atypical intestinal commensals in IL-21 receptor deficiency reshapes immune cell homeostasis and mucosal immunity. *Mucosal Immunol* 12**,** 85-96.

Cho, W.S., and Chae, C. (2003). Expression of inflammatory cytokines (TNF-alpha, IL-1, IL-6 and IL-8) in colon of pigs naturally infected with Salmonella typhimurium and S. choleraesuis. *J Vet Med A Physiol Pathol Clin Med* 50**,** 484-487.

Choi, S., Park, Y.S., Koga, T., Treloar, A., and Kim, K.C. (2011). TNF-alpha is a key regulator of MUC1, an anti-inflammatory molecule, during airway Pseudomonas aeruginosa infection. *Am J Respir Cell Mol Biol* 44**,** 255-260.

Choy, A.T.F., Carnevale, I., Coppola, S., Meijer, L.L., Kazemier, G., Zaura, E., Deng, D., and Giovannetti, E. (2018). The microbiome of pancreatic cancer: from molecular diagnostics to new therapeutic approaches to overcome chemoresistance caused by metabolic inactivation of gemcitabine. *Expert Rev Mol Diagn* 18**,** 1005-1009.

Chung, M., Zhao, N., Meier, R., Koestler, D.C., Wu, G., De Castillo, E., Paster, B.J., Charpentier, K., Izard, J., Kelsey, K.T., and Michaud, D.S. (2021). Comparisons of oral, intestinal, and pancreatic bacterial microbiomes in patients with pancreatic cancer and other gastrointestinal diseases. *J Oral Microbiol* 13**,** 1887680.

Cole, N., Bao, S., Willcox, M., and Husband, A.J. (1999). TNF-alpha production in the cornea in response to Pseudomonas aeruginosa challenge. *Immunol Cell Biol* 77**,** 164-166.

Collodel, G., Moretti, E., Campagna, M.S., Capitani, S., Lenzi, C., and Figura, N. (2010). Infection by CagA-positive Helicobacter pylori strains may contribute to alter the sperm quality of men with fertility disorders and increase the systemic levels of TNF-alpha. *Dig Dis Sci* 55**,** 94-100.

Colomb, F., Vidal, O., Bobowski, M., Krzewinski-Recchi, M.A., Harduin-Lepers, A., Mensier, E., Jaillard, S., Lafitte, J.J., Delannoy, P., and Groux-Degroote, S. (2014). TNF induces the expression of the sialyltransferase ST3Gal IV in human bronchial mucosa via MSK1/2 protein kinases and increases FliD/sialyl-Lewis(x)-mediated adhesion of Pseudomonas aeruginosa. *Biochem J* 457**,** 79-87.

Czepiel, J., Biesiada, G., Drozdz, M., Gdula-Argasinska, J., Zuranska, J., Marchewka, J., Perucki, W., Wolkow, P., and Garlicki, A. (2018). The presence of IL-8 +781 T/C polymorphism is associated with the parameters of severe Clostridium difficile infection. *Microb Pathog* 114**,** 281-285.

De Lima, C.D., Calegari-Silva, T.C., Pereira, R.M., Santos, S.A., Lopes, U.G., Plotkowski, M.C., and Saliba, A.M. (2012). ExoU activates NF-kappaB and increases IL-8/KC secretion during Pseudomonas aeruginosa infection. *PLoS One* 7**,** e41772.

De Martel, C., Llosa, A.E., Friedman, G.D., Vogelman, J.H., Orentreich, N., Stolzenberg-Solomon, R.Z., and Parsonnet, J. (2008). Helicobacter pylori infection and development of pancreatic cancer. *Cancer Epidemiol Biomarkers Prev* 17**,** 1188-1194.

Deharvengt, S., Wack, S., Aprahamian, M., and Hajri, A. (2005). Transcriptional tumor-selective promoter targeting of E. coli purine nucleoside phosphorylase for pancreatic cancer suicide gene therapy. *J Gene Med* 7**,** 672-680.

Dekita, M., Wu, Z., Ni, J., Zhang, X., Liu, Y., Yan, X., Nakanishi, H., and Takahashi, I. (2017). Cathepsin S Is Involved in Th17 Differentiation Through the Upregulation of IL-6 by Activating PAR-2 after Systemic Exposure to Lipopolysaccharide from Porphyromonas gingivalis. *Front Pharmacol* 8**,** 470.

Dey, I., and Bishayi, B. (2017). Role of Th17 and Treg cells in septic arthritis and the impact of the Th17/Treg -derived cytokines in the pathogenesis of S. aureus induced septic arthritis in mice. *Microb Pathog* 113**,** 248-264.

Dey, I., and Bishayi, B. (2020a). Impact of simultaneous neutralization of IL-17A and treatment with recombinant IL-2 on Th17-Treg cell population in S.aureus induced septic arthritis. *Microb Pathog* 139**,** 103903.

Dey, I., and Bishayi, B. (2020b). Role of different Th17 and Treg downstream signalling pathways in the pathogenesis of Staphylococcus aureus infection induced septic arthritis in mice. *Exp Mol Pathol* 116**,** 104485.

Diamantidis, M., Tsapournas, G., Kountouras, J., and Zavos, C. (2008). New aspects of regulatory signaling pathways and novel therapies in pancreatic cancer. *Curr Mol Med* 8**,** 12-37.

Diard, S., Lievin-Le Moal, V., Toribio, A.L., Boum, Y., Vigier, F., Servin, A.L., and Bouvet, O. (2009). Norepinephrine-dependently released Dr fimbriae of diffusely adhering Escherichia coli strain IH11128 promotes a mitogen-activated protein kinase ERK1/2-dependent production of pro-inflammatory cytokine, IL-8 in human intestinal Caco-2/TC7 cells. *Microbes Infect* 11**,** 886-894.

Ding, X., Jian, T., Li, J., Lv, H., Tong, B., Li, J., Meng, X., Ren, B., and Chen, J. (2020). Chicoric Acid Ameliorates Nonalcoholic Fatty Liver Disease via the AMPK/Nrf2/NFkappaB Signaling Pathway and Restores Gut Microbiota in High-Fat-Diet-Fed Mice. *Oxid Med Cell Longev* 2020**,** 9734560.

Diya, Z., Lili, C., Shenglai, L., Zhiyuan, G., and Jie, Y. (2008). Lipopolysaccharide (LPS) of Porphyromonas gingivalis induces IL-1beta, TNF-alpha and IL-6 production by THP-1 cells in a way different from that of Escherichia coli LPS. *Innate Immun* 14**,** 99-107.

Dos Santos, S.A., De Andrade Junior, D.R., and De Andrade, D.R. (2011). TNF-alpha production and apoptosis in hepatocytes after Listeria monocytogenes and Salmonella Typhimurium invasion. *Rev Inst Med Trop Sao Paulo* 53**,** 107-112.

Duan, C., Kuang, L., Xiang, X., Zhang, J., Zhu, Y., Wu, Y., Yan, Q., Liu, L., and Li, T. (2020). Activated Drp1-mediated mitochondrial ROS influence the gut microbiome and intestinal barrier after hemorrhagic shock. *Aging (Albany NY)* 12**,** 1397-1416.

Dutta, P., and Bishayi, B. (2021). Neutralization of TNF-alpha and IL-1beta Regulates CXCL8 Production through CXCL8/CXCR1 Axis in Macrophages during Staphylococcus aureus Infection. *Immunol Invest* 50**,** 700-725.

Ebelt, N.D., Zamloot, V., Zuniga, E., Passi, K.B., Sobocinski, L.J., Young, C.A., Blazar, B.R., and Manuel, E.R. (2021). Collagenase-Expressing Salmonella Targets Major Collagens in Pancreatic Cancer Leading to Reductions in Immunosuppressive Subsets and Tumor Growth. *Cancers (Basel)* 13.

Elatrech, I., Marzaioli, V., Boukemara, H., Bournier, O., Neut, C., Darfeuille-Michaud, A., Luis, J., Dubuquoy, L., El-Benna, J., My-Chan Dang, P., and Marie, J.C. (2015). Escherichia coli LF82 differentially regulates ROS production and mucin expression in intestinal epithelial T84 cells: implication of NOX1. *Inflamm Bowel Dis* 21**,** 1018-1026.

Engevik, M.A., Danhof, H.A., Ruan, W., Engevik, A.C., Chang-Graham, A.L., Engevik, K.A., Shi, Z., Zhao, Y., Brand, C.K., and Krystofiak, E.S. (2021). Fusobacterium nucleatum secretes outer membrane vesicles and promotes intestinal inflammation. *MBio* 12**,** e02706-02720.

Engevik, M.A., Danhof, H.A., Shrestha, R., Chang-Graham, A.L., Hyser, J.M., Haag, A.M., Mohammad, M.A., Britton, R.A., Versalovic, J., and Sorg, J.A. (2020). Reuterin disrupts Clostridioides difficile metabolism and pathogenicity through reactive oxygen species generation. *Gut Microbes* 12**,** 1795388.

Ertz-Archambault, N., Keim, P., and Von Hoff, D. (2017). Microbiome and pancreatic cancer: A comprehensive topic review of literature. *World J Gastroenterol* 23**,** 1899-1908.

Evoy, D., Hirschowitz, E.A., Naama, H.A., Li, X.K., Crystal, R.G., Daly, J.M., and Lieberman, M.D. (1997). In vivo adenoviral-mediated gene transfer in the treatment of pancreatic cancer. *J Surg Res* 69**,** 226-231.

Fan, L., Wang, Q., De La Fuente-Nunez, C., Sun, F.J., Xia, J.G., Xia, P.Y., and Hancock, R.E. (2014). Increased IL-8 production in human bronchial epithelial cells after exposure to azithromycin-pretreated Pseudomonas aeruginosa in vitro. *FEMS Microbiol Lett* 355**,** 43-50.

Fan, X., Alekseyenko, A.V., Wu, J., Peters, B.A., Jacobs, E.J., Gapstur, S.M., Purdue, M.P., Abnet, C.C., Stolzenberg-Solomon, R., Miller, G., Ravel, J., Hayes, R.B., and Ahn, J. (2018). Human oral microbiome and prospective risk for pancreatic cancer: a population-based nested case-control study. *Gut* 67**,** 120-127.

Felix, K., Hauck, O., Fritz, S., Hinz, U., Schnolzer, M., Kempf, T., Warnken, U., Michel, A., Pawlita, M., and Werner, J. (2013). Serum protein signatures differentiating autoimmune pancreatitis versus pancreatic cancer. *PLoS One* 8**,** e82755.

Ferrante, A., Martin, A.J., Bates, E.J., Kowanko, I.C., Harvey, D.P., Parsons, D., Rathjen, D.A., Russ, G., and Dayer, J.M. (1994). Interaction of Staphylococcus aureus with human neutrophils and the down-regulation of TNF receptors. *J Immunol* 152**,** 3998-4004.

Figueiredo, R.D.A., Ortega, A.C., Gonzalez Maldonado, L.A., Castro, R.D., Avila-Campos, M.J., Rossa, C., and Aquino, S.G. (2020). Perillyl alcohol has antibacterial effects and reduces ROS production in macrophages. *J Appl Oral Sci* 28**,** e20190519.

Flaherty, R.A., Aronoff, D.M., Gaddy, J.A., Petroff, M.G., and Manning, S.D. (2021). Distinct Group B Streptococcus Sequence and Capsule Types Differentially Impact Macrophage Stress and Inflammatory Signaling Responses. *Infect Immun* 89.

Flaherty, R.A., Borges, E.C., Sutton, J.A., Aronoff, D.M., Gaddy, J.A., Petroff, M.G., and Manning, S.D. (2019). Genetically distinct Group B Streptococcus strains induce varying macrophage cytokine responses. *PLoS One* 14**,** e0222910.

Flores-Maldonado, O., Gonzalez, G.M., Montoya, A., Andrade, A., Trevino-Rangel, R., Donis-Maturano, L., Tavares-Carreon, F., and Becerril-Garcia, M.A. (2022). Dissemination of Gram-positive bacteria to the lung of newborn mice increases local IL-6 and TNFalpha levels in lethal bacteremia. *Microbes Infect***,** 104984.

Foschetti, D.A., Braga-Neto, M.B., Bolick, D., Moore, J., Alves, L.A., Martins, C.S., Bomfin, L.E., Santos, A., Leitao, R., Brito, G., and Warren, C.A. (2020). Clostridium difficile toxins or infection induce upregulation of adenosine receptors and IL-6 with early pro-inflammatory and late anti-inflammatory pattern. *Braz J Med Biol Res* 53**,** e9877.

Gaida, M.M., Mayer, C., Dapunt, U., Stegmaier, S., Schirmacher, P., Wabnitz, G.H., and Hansch, G.M. (2016). Expression of the bitter receptor T2R38 in pancreatic cancer: localization in lipid droplets and activation by a bacteria-derived quorum-sensing molecule. *Oncotarget* 7**,** 12623-12632.

Gaignage, M., Zhang, X., Stockis, J., Dedobbeleer, O., Michiels, C., Cochez, P., Dumoutier, L., Coulie, P.G., and Lucas, S. (2022). Blocking GARP-mediated activation of TGF-beta1 did not alter innate or adaptive immune responses to bacterial infection or protein immunization in mice. *Cancer Immunol Immunother*.

Gaiser, R.A., Halimi, A., Alkharaan, H., Lu, L., Davanian, H., Healy, K., Hugerth, L.W., Ateeb, Z., Valente, R., Fernandez Moro, C., Del Chiaro, M., and Sallberg Chen, M. (2019). Enrichment of oral microbiota in early cystic precursors to invasive pancreatic cancer. *Gut* 68**,** 2186-2194.

Galanakis, E., Di Cello, F., Paul-Satyaseela, M., and Kim, K.S. (2006). Escherichia coli K1 induces IL-8 expression in human brain microvascular endothelial cells. *Eur Cytokine Netw* 17**,** 260-265.

Galdiero, M., D'isanto, M., Vitiello, M., Finamore, E., Peluso, L., and Galdiero, M. (2001). Porins from Salmonella enterica serovar Typhimurium induce TNF-alpha, IL-6 and IL-8 release by CD14-independent and CD11a/CD18-dependent mechanisms. *Microbiology (Reading)* 147**,** 2697-2704.

Gambari, R., Borgatti, M., Bezzerri, V., Nicolis, E., Lampronti, I., Dechecchi, M.C., Mancini, I., Tamanini, A., and Cabrini, G. (2010). Decoy oligodeoxyribonucleotides and peptide nucleic acids-DNA chimeras targeting nuclear factor kappa-B: inhibition of IL-8 gene expression in cystic fibrosis cells infected with Pseudomonas aeruginosa. *Biochem Pharmacol* 80**,** 1887-1894.

Gao, M., Li, D., Hu, Y., Zhang, Y., Zou, Q., and Wang, D.C. (2012). Crystal structure of TNF-alpha-inducing protein from Helicobacter pylori in active form reveals the intrinsic molecular flexibility for unique DNA-binding. *PLoS One* 7**,** e41871.

Gawin, A., Wex, T., Lawniczak, M., Malfertheiner, P., and Starzynska, T. (2012). [Helicobacter pylori infection in pancreatic cancer]. *Pol Merkur Lekarski* 32**,** 103-107.

Genton, L., Lazarevic, V., Stojanovic, O., Spiljar, M., Djaafar, S., Koessler, T., Dutoit, V., Gaia, N., Mareschal, J., Macpherson, A.J., Herrmann, F., Trajkovski, M., and Schrenzel, J. (2021). Metataxonomic and Metabolic Impact of Fecal Microbiota Transplantation From Patients With Pancreatic Cancer Into Germ-Free Mice: A Pilot Study. *Front Cell Infect Microbiol* 11**,** 752889.

Gerstel, U., Czapp, M., Bartels, J., and Schroder, J.M. (2009). Rhamnolipid-induced shedding of flagellin from Pseudomonas aeruginosa provokes hBD-2 and IL-8 response in human keratinocytes. *Cell Microbiol* 11**,** 842-853.

Ghasemi, M., Khorsandi, K., and Kianmehr, Z. (2021). Photodynamic inactivation with curcumin and silver nanoparticles hinders Pseudomonas aeruginosa planktonic and biofilm formation: evaluation of glutathione peroxidase activity and ROS production. *World J Microbiol Biotechnol* 37**,** 149.

Gholam-Mostafaei, F.S., Yadegar, A., Aghdaei, H.A., Azimirad, M., Daryani, N.E., and Zali, M.R. (2020). Anti-TNF containing regimens may be associated with increased risk of Clostridioides difficile infection in patients with underlying inflammatory bowel disease. *Curr Res Transl Med* 68**,** 125-130.

Giantonio, B.J., Alpaugh, R.K., Schultz, J., Mcaleer, C., Newton, D.W., Shannon, B., Guedez, Y., Kotb, M., Vitek, L., Persson, R., Gunnarsson, P.O., Kalland, T., Dohlsten, M., Persson, B., and Weiner, L.M. (1997). Superantigen-based immunotherapy: a phase I trial of PNU-214565, a monoclonal antibody-staphylococcal enterotoxin A recombinant fusion protein, in advanced pancreatic and colorectal cancer. *J Clin Oncol* 15**,** 1994-2007.

Glowczyk, I., Wong, A., Potempa, B., Babyak, O., Lech, M., Lamont, R.J., Potempa, J., and Koziel, J. (2017). Inactive Gingipains from P. gingivalis Selectively Skews T Cells toward a Th17 Phenotype in an IL-6 Dependent Manner. *Front Cell Infect Microbiol* 7**,** 140.

Gobert, A.P., and Wilson, K.T. (2017). Polyamine- and NADPH-dependent generation of ROS during Helicobacter pylori infection: A blessing in disguise. *Free Radic Biol Med* 105**,** 16-27.

Godaly, G., Proudfoot, A.E., Offord, R.E., Svanborg, C., and Agace, W.W. (1997). Role of epithelial interleukin-8 (IL-8) and neutrophil IL-8 receptor A in Escherichia coli-induced transuroepithelial neutrophil migration. *Infect Immun* 65**,** 3451-3456.

Gomez-Rubio, P., Rosato, V., Marquez, M., Bosetti, C., Molina-Montes, E., Rava, M., Pinero, J., Michalski, C.W., Farre, A., Molero, X., Lohr, M., Ilzarbe, L., Perea, J., Greenhalf, W., O'rorke, M., Tardon, A., Gress, T., Barbera, V.M., Crnogorac-Jurcevic, T., Munoz-Bellvis, L., Dominguez-Munoz, E., Gutierrez-Sacristan, A., Balsells, J., Costello, E., Guillen-Ponce, C., Huang, J., Iglesias, M., Kleeff, J., Kong, B., Mora, J., Murray, L., O'driscoll, D., Pelaez, P., Poves, I., Lawlor, R.T., Carrato, A., Hidalgo, M., Scarpa, A., Sharp, L., Furlong, L.I., Real, F.X., La Vecchia, C., Malats, N., and Pangen, E.U.S.I. (2017). A systems approach identifies time-dependent associations of multimorbidities with pancreatic cancer risk. *Ann Oncol* 28**,** 1618-1624.

Gong, Y., Chen, S., Fu, Y., Liu, Y., Wang, Y., Yang, H., Liu, H., and Tang, L. (2021). MUC4 is a novel mediator in H. pylori infection-related pancreatic cancer. *Oncol Lett* 21**,** 123.

Govindan, J.A., Jayamani, E., and Ruvkun, G. (2019). ROS-based lethality of Caenorhabditis elegans mitochondrial electron transport mutants grown on Escherichia coli siderophore iron release mutants. *Proc Natl Acad Sci U S A* 116**,** 21651-21658.

Grassl, G.A., Kracht, M., Wiedemann, A., Hoffmann, E., Aepfelbacher, M., Von Eichel-Streiber, C., Bohn, E., and Autenrieth, I.B. (2003). Activation of NF-kappaB and IL-8 by Yersinia enterocolitica invasin protein is conferred by engagement of Rac1 and MAP kinase cascades. *Cell Microbiol* 5**,** 957-971.

Green, N.K., Youngs, D.J., Neoptolemos, J.P., Friedlos, F., Knox, R.J., Springer, C.J., Anlezark, G.M., Michael, N.P., Melton, R.G., Ford, M.J., Young, L.S., Kerr, D.J., and Searle, P.F. (1997). Sensitization of colorectal and pancreatic cancer cell lines to the prodrug 5-(aziridin-1-yl)-2,4-dinitrobenzamide (CB1954) by retroviral transduction and expression of the E. coli nitroreductase gene. *Cancer Gene Ther* 4**,** 229-238.

Gregg, J.A. (1977). Detection of bacterial infection of the pancreatic ducts in patients with pancreatitis and pancreatic cancer during endoscopic cannulation of the pancreatic duct. *Gastroenterology* 73**,** 1005-1007.

Guo, F., Tang, J., Zhou, Z., Dou, Y., Van Lonkhuyzen, D., Gao, C., and Huan, J. (2012). GEF-H1-RhoA signaling pathway mediates LPS-induced NF-kappaB transactivation and IL-8 synthesis in endothelial cells. *Mol Immunol* 50**,** 98-107.

Guo, Y., Liu, W., and Wu, J. (2016). Helicobacter pylori infection and pancreatic cancer risk: A meta-analysis. *J Cancer Res Ther* 12**,** C229-C232.

Halimi, A., Gabarrini, G., Sobkowiak, M.J., Ateeb, Z., Davanian, H., Gaiser, R.A., Arnelo, U., Valente, R., Wong, A.Y.W., Moro, C.F., Del Chiaro, M., Ozenci, V., and Chen, M.S. (2021). Isolation of pancreatic microbiota from cystic precursors of pancreatic cancer with intracellular growth and DNA damaging properties. *Gut Microbes* 13**,** 1983101.

Hamajima, N., Shibata, A., Katsuda, N., Matsuo, K., Ito, H., Saito, T., Tajima, K., and Tominaga, S. (2003). Subjects with TNF-A-857TT and -1031TT genotypes showed the highest Helicobacter pylori seropositive rate compared with those with other genotypes. *Gastric Cancer* 6**,** 230-236.

Han, L., Ma, Q., Yu, J., Gong, Z., Ma, C., Xu, Y., Deng, G., and Wu, X. (2020). Autophagy plays a protective role during Pseudomonas aeruginosa-induced apoptosis via ROS-MAPK pathway. *Innate Immun* 26**,** 580-591.

Handa, O., Naito, Y., and Yoshikawa, T. (2010). Helicobacter pylori: a ROS-inducing bacterial species in the stomach. *Inflamm Res* 59**,** 997-1003.

Hansen, A., Alston, L., Tulk, S.E., Schenck, L.P., Grassie, M.E., Alhassan, B.F., Veermalla, A.T., Al-Bashir, S., Gendron, F.P., Altier, C., Macdonald, J.A., Beck, P.L., and Hirota, S.A. (2013). The P2Y6 receptor mediates Clostridium difficile toxin-induced CXCL8/IL-8 production and intestinal epithelial barrier dysfunction. *PLoS One* 8**,** e81491.

Harada, A., Sekido, N., Kuno, K., Akiyama, M., Kasahara, T., Nakanishi, I., Mukaida, N., and Matsushima, K. (1993). Expression of recombinant rabbit IL-8 in Escherichia coli and establishment of the essential involvement of IL-8 in recruiting neutrophils into lipopolysaccharide-induced inflammatory site of rabbit skin. *Int Immunol* 5**,** 681-690.

Harder, J., Meyer-Hoffert, U., Teran, L.M., Schwichtenberg, L., Bartels, J., Maune, S., and Schroder, J.M. (2000). Mucoid Pseudomonas aeruginosa, TNF-alpha, and IL-1beta, but not IL-6, induce human beta-defensin-2 in respiratory epithelia. *Am J Respir Cell Mol Biol* 22**,** 714-721.

Hart, A.R., Kennedy, H., and Harvey, I. (2008). Pancreatic cancer: a review of the evidence on causation. *Clin Gastroenterol Hepatol* 6**,** 275-282.

Hayakawa, S., Kawamura, M., Sato, T., Hirano, T., Kikuchi, T., Watanabe, A., and Fujimura, S. (2019). An alpha-Lipoic acid derivative, and anti-ROS agent, prevents the acquisition of multi-drug resistance in clinical isolates of Pseudomonas aeruginosa. *J Infect Chemother* 25**,** 28-33.

Haziot, A., Hijiya, N., Schultz, K., Zhang, F., Gangloff, S.C., and Goyert, S.M. (1999). CD14 plays no major role in shock induced by Staphylococcus aureus but down-regulates TNF-alpha production. *J Immunol* 162**,** 4801-4805.

He, D., Sougioultzis, S., Hagen, S., Liu, J., Keates, S., Keates, A.C., Pothoulakis, C., and Lamont, J.T. (2002). Clostridium difficile toxin A triggers human colonocyte IL-8 release via mitochondrial oxygen radical generation. *Gastroenterology* 122**,** 1048-1057.

He, X.P., Li, Z.S., Zhu, R.M., Tu, Z.X., Gao, J., Pan, X., Gong, Y.F., Jin, J., Man, X.H., Wu, H.Y., and Xu, A.F. (2006). Effects of recombinant human canstatin protein in the treatment of pancreatic cancer. *World J Gastroenterol* 12**,** 6652-6657.

Hedges, S.R., Bjarnadottir, M., Agace, W., Hang, L., and Svanborg, C. (1996). Immunoregulatory cytokines modify Escherichia coli induced uroepithelial cell IL-6 and IL-8 responses. *Cytokine* 8**,** 686-697.

Heller, J.J., Schjerven, H., Li, S., Lee, A., Qiu, J., Chen, Z.M., Smale, S.T., and Zhou, L. (2014). Restriction of IL-22-producing T cell responses and differential regulation of regulatory T cell compartments by zinc finger transcription factor Ikaros. *J Immunol* 193**,** 3934-3946.

Hellmig, S., Bartscht, T., Fischbach, W., Folsch, U.R., and Schreiber, S. (2008). Interleukin-10 (-819 C/T) and TNF-A (-308 G/A) as risk factors for H. pylori-associated gastric MALT-lymphoma. *Dig Dis Sci* 53**,** 2007-2008.

Herath, T.D., Wang, Y., Seneviratne, C.J., Lu, Q., Darveau, R.P., Wang, C.Y., and Jin, L. (2011). Porphyromonas gingivalis lipopolysaccharide lipid A heterogeneity differentially modulates the expression of IL-6 and IL-8 in human gingival fibroblasts. *J Clin Periodontol* 38**,** 694-701.

Hickling, T.P., Sim, R.B., and Malhotra, R. (1998). Induction of TNF-alpha release from human buffy coat cells by Pseudomonas aeruginosa is reduced by lung surfactant protein A. *FEBS Lett* 437**,** 65-69.

Hinshaw, L.B., Emerson, T.E., Jr., Taylor, F.B., Jr., Chang, A.C., Duerr, M., Peer, G.T., Flournoy, D.J., White, G.L., Kosanke, S.D., Murray, C.K., and Et Al. (1992). Lethal Staphylococcus aureus-induced shock in primates: prevention of death with anti-TNF antibody. *J Trauma* 33**,** 568-573.

Hippenstiel, S., Schmeck, B., Seybold, J., Krull, M., Eichel-Streiber, C., and Suttorp, N. (2002). Reduction of tumor necrosis factor-alpha (TNF-alpha) related nuclear factor-kappaB (NF-kappaB) translocation but not inhibitor kappa-B (Ikappa-B)-degradation by Rho protein inhibition in human endothelial cells. *Biochem Pharmacol* 64**,** 971-977.

Hirabayashi, M., Inoue, M., Sawada, N., Saito, E., Abe, S.K., Hidaka, A., Iwasaki, M., Yamaji, T., Shimazu, T., and Tsugane, S. (2019). Helicobacter pylori infection, atrophic gastritis, and risk of pancreatic cancer: A population-based cohort study in a large Japanese population: the JPHC Study. *Sci Rep* 9**,** 6099.

Hirabayashi, M., Inoue, M., Sawada, N., Saito, E., Abe, S.K., Hidaka, A., Iwasaki, M., Yamaji, T., Shimazu, T., and Tsugane, S. (2020). Author Correction: Helicobacter pylori infection, atrophic gastritis, and risk of pancreatic cancer: A population-based cohort study in a large Japanese population: the JPHC Study. *Sci Rep* 10**,** 12950.

Hirai, K., Fujimura, S., Shibata, Y., Ishihara, K., Kato, T., Okuda, K., and Nakamura, T. (2000). Differences in TNF-alpha producing activity from murine peritoneal macrophages induced by lipopolysaccharides of Prevotella heparinolytica and Porphyromonas gingivalis. *Bull Tokyo Dent Coll* 41**,** 135-140.

Hiraki, D., Uehara, O., Kuramitsu, Y., Morikawa, T., Harada, F., Yoshida, K., Akino, K., Chiba, I., Asaka, M., and Abiko, Y. (2020). P. gingivalis Lipopolysaccharide Stimulates the Upregulated Expression of the Pancreatic Cancer-Related Genes Regenerating Islet-Derived 3 A/G in Mouse Pancreas. *Int J Mol Sci* 21.

Hirata, Y., Ohmae, T., Shibata, W., Maeda, S., Ogura, K., Yoshida, H., Kawabe, T., and Omata, M. (2006). MyD88 and TNF receptor-associated factor 6 are critical signal transducers in Helicobacter pylori-infected human epithelial cells. *J Immunol* 176**,** 3796-3803.

Hiroshima, Y., Zhang, Y., Murakami, T., Maawy, A., Miwa, S., Yamamoto, M., Yano, S., Sato, S., Momiyama, M., Mori, R., Matsuyama, R., Chishima, T., Tanaka, K., Ichikawa, Y., Bouvet, M., Endo, I., Zhao, M., and Hoffman, R.M. (2014a). Efficacy of tumor-targeting Salmonella typhimurium A1-R in combination with anti-angiogenesis therapy on a pancreatic cancer patient-derived orthotopic xenograft (PDOX) and cell line mouse models. *Oncotarget* 5**,** 12346-12357.

Hiroshima, Y., Zhao, M., Maawy, A., Zhang, Y., Katz, M.H., Fleming, J.B., Uehara, F., Miwa, S., Yano, S., Momiyama, M., Suetsugu, A., Chishima, T., Tanaka, K., Bouvet, M., Endo, I., and Hoffman, R.M. (2014b). Efficacy of Salmonella typhimurium A1-R versus chemotherapy on a pancreatic cancer patient-derived orthotopic xenograft (PDOX). *J Cell Biochem* 115**,** 1254-1261.

Hiroshima, Y., Zhao, M., Zhang, Y., Maawy, A., Hassanein, M.K., Uehara, F., Miwa, S., Yano, S., Momiyama, M., Suetsugu, A., Chishima, T., Tanaka, K., Bouvet, M., Endo, I., and Hoffman, R.M. (2013). Comparison of efficacy of Salmonella typhimurium A1-R and chemotherapy on stem-like and non-stem human pancreatic cancer cells. *Cell Cycle* 12**,** 2774-2780.

Hodgkinson, L. (2010). Digestive Disease Week 2010. Turning Science into Medicine--part 2. *IDrugs* 13**,** 424-426.

Hoffman, R.M., and Bouvet, M. (2015). Nanoparticle albumin-bound-paclitaxel: a limited improvement under the current therapeutic paradigm of pancreatic cancer. *Expert Opin Pharmacother* 16**,** 943-947.

Holden, J.A., Attard, T.J., Laughton, K.M., Mansell, A., O'brien-Simpson, N.M., and Reynolds, E.C. (2014). Porphyromonas gingivalis lipopolysaccharide weakly activates M1 and M2 polarized mouse macrophages but induces inflammatory cytokines. *Infection and immunity* 82**,** 4190-4203.

Hollevoet, K., Mason-Osann, E., Muller, F., and Pastan, I. (2015). Methylation-associated partial down-regulation of mesothelin causes resistance to anti-mesothelin immunotoxins in a pancreatic cancer cell line. *PLoS One* 10**,** e0122462.

Hong, G., Davies, C., Omole, Z., Liaw, J., Grabowska, A.D., Canonico, B., Corcionivoschi, N., Wren, B., Dorrell, N., and Elmi, A. (2022). Campylobacter jejuni modulates reactive oxygen species production and NADPH oxidase 1 expression in human intestinal epithelial cell. *bioRxiv*.

Howe, C., Mitchell, J., Kim, S.J., Im, E., and Rhee, S.H. (2019). Pten gene deletion in intestinal epithelial cells enhances susceptibility to Salmonella Typhimurium infection in mice. *J Microbiol* 57**,** 1012-1018.

Htwe, P., Aung, H., Kywe, B., Niang, P.T., Oo, T.S., Monhandas, S., Kelly, L., and Goldman, D.L. (2021). Endotoxin acts synergistically with C. difficile toxin B to increase IL-1 β production: a potential role for the intestinal biome in modifying the severity of C. difficile colitis. *The Journal of Infectious Diseases*.

Hu, C., Yan, Y., Ji, F., and Zhou, H. (2021). Maternal Obesity Increases Oxidative Stress in Placenta and It Is Associated With Intestinal Microbiota. *Front Cell Infect Microbiol* 11**,** 671347.

Huang, F.C., Lu, Y.T., and Liao, Y.H. (2020). Beneficial effect of probiotics on Pseudomonas aeruginosa-infected intestinal epithelial cells through inflammatory IL-8 and antimicrobial peptide human beta-defensin-2 modulation. *Innate Immun* 26**,** 592-600.

Huang, J., Zagai, U., Hallmans, G., Nyren, O., Engstrand, L., Stolzenberg-Solomon, R., Duell, E.J., Overvad, K., Katzke, V.A., Kaaks, R., Jenab, M., Park, J.Y., Murillo, R., Trichopoulou, A., Lagiou, P., Bamia, C., Bradbury, K.E., Riboli, E., Aune, D., Tsilidis, K.K., Capella, G., Agudo, A., Krogh, V., Palli, D., Panico, S., Weiderpass, E., Tjonneland, A., Olsen, A., Martinez, B., Redondo-Sanchez, D., Chirlaque, M.D., Hm Peeters, P., Regner, S., Lindkvist, B., Naccarati, A., Ardanaz, E., Larranaga, N., Boutron-Ruault, M.C., Rebours, V., Barre, A., Bueno-De-Mesquita, H.B., and Ye, W. (2017). Helicobacter pylori infection, chronic corpus atrophic gastritis and pancreatic cancer risk in the European Prospective Investigation into Cancer and Nutrition (EPIC) cohort: A nested case-control study. *Int J Cancer* 140**,** 1727-1735.

Hubbard, L.L., Wilke, C.A., White, E.S., and Moore, B.B. (2011). PTEN limits alveolar macrophage function against Pseudomonas aeruginosa after bone marrow transplantation. *Am J Respir Cell Mol Biol* 45**,** 1050-1058.

Hultgren, O., Eugster, H.P., Sedgwick, J.D., Korner, H., and Tarkowski, A. (1998). TNF/lymphotoxin-alpha double-mutant mice resist septic arthritis but display increased mortality in response to Staphylococcus aureus. *J Immunol* 161**,** 5937-5942.

Hyun, I.K., Lee, J.S., Yoon, J.W., and Kang, S.S. (2021). Skimmed milk fermented by lactic acid bacteria inhibits adipogenesis in 3T3-L1 pre-adipocytes by downregulating PPARgamma via TNF-alpha induction in vitro. *Food Funct* 12**,** 8605-8614.

Ianovich, O.O., Nosova, E.S., and Titov, L.P. (2013). [Polymorphism of the genes IL-1RA and TNF-alpha in patients with gastritis and duodenal ulcer associated with Helicobacter pylori]. *Mol Gen Mikrobiol Virusol***,** 31-34.

Im, J., Baik, J.E., Kim, K.W., Kang, S.S., Jeon, J.H., Park, O.J., Kim, H.Y., Kum, K.Y., Yun, C.H., and Han, S.H. (2015). Enterococcus faecalis lipoteichoic acid suppresses Aggregatibacter actinomycetemcomitans lipopolysaccharide-induced IL-8 expression in human periodontal ligament cells. *Int Immunol* 27**,** 381-391.

Inomata, M., Into, T., and Murakami, Y. (2010). Suppressive effect of the antimicrobial peptide LL-37 on expression of IL-6, IL-8 and CXCL10 induced by Porphyromonas gingivalis cells and extracts in human gingival fibroblasts. *Eur J Oral Sci* 118**,** 574-581.

Ishida, Y., Goto, Y., Kondo, T., Kurata, M., Nishio, K., Kawai, S., Osafune, T., Naito, M., and Hamajima, N. (2006). Eradication rate of Helicobacter pylori according to genotypes of CYP2C19, IL-1B, and TNF-A. *Int J Med Sci* 3**,** 135-140.

Jacob, J.A. (2016). Study Links Periodontal Disease Bacteria to Pancreatic Cancer Risk. *JAMA* 315**,** 2653-2654.

Jain, U., Cao, Q., Thomas, N.A., Woodruff, T.M., Schwaeble, W.J., Stover, C.M., and Stadnyk, A.W. (2015). Properdin provides protection from citrobacter rodentium–induced intestinal inflammation in a C5a/IL-6–dependent manner. *The Journal of Immunology* 194**,** 3414-3421.

Jang, J.Y., Yoon, H.J., Yoon, J.Y., Kim, H.S., Lee, S.J., Kim, K.H., Kim, D.J., Jang, S., Han, B.G., Lee, B.I., and Suh, S.W. (2009). Crystal structure of the TNF-alpha-Inducing protein (Tipalpha) from Helicobacter pylori: Insights into Its DNA-binding activity. *J Mol Biol* 392**,** 191-197.

Janssen, R., Van Wengen, A., Verhard, E., De Boer, T., Zomerdijk, T., Ottenhoff, T.H., and Van Dissel, J.T. (2002). Divergent role for TNF-alpha in IFN-gamma-induced killing of Toxoplasma gondii and Salmonella typhimurium contributes to selective susceptibility of patients with partial IFN-gamma receptor 1 deficiency. *J Immunol* 169**,** 3900-3907.

Jefferson, K.K., Smith, M.F., Jr., and Bobak, D.A. (1999). Roles of intracellular calcium and NF-kappa B in the Clostridium difficile toxin A-induced up-regulation and secretion of IL-8 from human monocytes. *J Immunol* 163**,** 5183-5191.

Jeffrey, M.P., Macpherson, C.W., Mathieu, O., Tompkins, T.A., and Green-Johnson, J.M. (2020). Secretome-Mediated Interactions with Intestinal Epithelial Cells: A Role for Secretome Components from Lactobacillus rhamnosus R0011 in the Attenuation of Salmonella enterica Serovar Typhimurium Secretome and TNF-alpha-Induced Proinflammatory Responses. *J Immunol* 204**,** 2523-2534.

Jin, M., Lu, J., Chen, Z., Nguyen, S.H., Mao, L., Li, J., Yuan, Z., and Guo, J. (2018). Antidepressant fluoxetine induces multiple antibiotics resistance in Escherichia coli via ROS-mediated mutagenesis. *Environ Int* 120**,** 421-430.

Jin, W., Ibeagha-Awemu, E.M., Liang, G., Beaudoin, F., Zhao, X., and Guan Le, L. (2014). Transcriptome microRNA profiling of bovine mammary epithelial cells challenged with Escherichia coli or Staphylococcus aureus bacteria reveals pathogen directed microRNA expression profiles. *BMC Genomics* 15**,** 181.

Jo, A.R., Baek, K.J., Shin, J.E., and Choi, Y. (2014). Mechanisms of IL-8 suppression by Treponema denticola in gingival epithelial cells. *Immunol Cell Biol* 92**,** 139-147.

Johansen, D., Manjer, J., Regner, S., and Lindkvist, B. (2010). Pre-diagnostic levels of anionic trypsinogen, cationic trypsinogen, and pancreatic secretory trypsin inhibitor in relation to pancreatic cancer risk. *Pancreatology* 10**,** 229-237.

Jones, M.M., Vanyo, S.T., and Visser, M.B. (2019). The Msp Protein of Treponema denticola Interrupts Activity of Phosphoinositide Processing in Neutrophils. *Infect Immun* 87.

Joseph, J., Ametepe, E.S., Haribabu, N., Agbayani, G., Krishnan, L., Blais, A., and Sad, S. (2016). Inhibition of ROS and upregulation of inflammatory cytokines by FoxO3a promotes survival against Salmonella typhimurium. *Nat Commun* 7**,** 12748.

Joseph, T., Look, D., and Ferkol, T. (2005). NF-kappaB activation and sustained IL-8 gene expression in primary cultures of cystic fibrosis airway epithelial cells stimulated with Pseudomonas aeruginosa. *Am J Physiol Lung Cell Mol Physiol* 288**,** L471-479.

Jouneau, S., Bonizec, M., Belleguic, C., Desrues, B., Brinchault, G., Galaine, J., Gangneux, J.P., and Martin-Chouly, C. (2011). Anti-inflammatory effect of fluvastatin on IL-8 production induced by Pseudomonas aeruginosa and Aspergillus fumigatus antigens in cystic fibrosis. *PLoS One* 6**,** e22655.

Kabwe, M., Meehan-Andrews, T., Ku, H., Petrovski, S., Batinovic, S., Chan, H.T., and Tucci, J. (2021). Lytic Bacteriophage EFA1 Modulates HCT116 Colon Cancer Cell Growth and Upregulates ROS Production in an Enterococcus faecalis Co-culture System. *Front Microbiol* 12**,** 650849.

Kaliberova, L.N., Della Manna, D.L., Krendelchtchikova, V., Black, M.E., Buchsbaum, D.J., and Kaliberov, S.A. (2008). Molecular chemotherapy of pancreatic cancer using novel mutant bacterial cytosine deaminase gene. *Mol Cancer Ther* 7**,** 2845-2854.

Kang, S.S., Noh, S.Y., Park, O.J., Yun, C.H., and Han, S.H. (2015). Staphylococcus aureus induces IL-8 expression through its lipoproteins in the human intestinal epithelial cell, Caco-2. *Cytokine* 75**,** 174-180.

Kang, W., Jia, Z., Tang, D., Zhang, Z., Gao, H., He, K., and Feng, Q. (2019). Fusobacterium nucleatum Facilitates Apoptosis, ROS Generation, and Inflammatory Cytokine Production by Activating AKT/MAPK and NF-kappaB Signaling Pathways in Human Gingival Fibroblasts. *Oxid Med Cell Longev* 2019**,** 1681972.

Kapetanovic, R., Parlato, M., Fitting, C., Quesniaux, V., Cavaillon, J.M., and Adib-Conquy, M. (2011). Mechanisms of TNF induction by heat-killed Staphylococcus aureus differ upon the origin of mononuclear phagocytes. *Am J Physiol Cell Physiol* 300**,** C850-859.

Karlsson, I., Hagman, R., Guo, Y., Humblot, P., Wang, L., and Wernersson, S. (2015). Pathogenic Escherichia coli and lipopolysaccharide enhance the expression of IL-8, CXCL5, and CXCL10 in canine endometrial stromal cells. *Theriogenology* 84**,** 34-42.

Karpinski, T.M. (2019). The Microbiota and Pancreatic Cancer. *Gastroenterol Clin North Am* 48**,** 447-464.

Kato, Y., Hagiwara, M., Ishihara, Y., Isoda, R., Sugiura, S., Komatsu, T., Ishida, N., Noguchi, T., and Matsushita, K. (2014). TNF-alpha augmented Porphyromonas gingivalis invasion in human gingival epithelial cells through Rab5 and ICAM-1. *BMC Microbiol* 14**,** 229.

Kawaguchi, K., Miyake, K., Zhao, M., Kiyuna, T., Igarashi, K., Miyake, M., Higuchi, T., Oshiro, H., Bouvet, M., Unno, M., and Hoffman, R.M. (2018). Tumor targeting Salmonella typhimurium A1-R in combination with gemcitabine (GEM) regresses partially GEM-resistant pancreatic cancer patient-derived orthotopic xenograft (PDOX) nude mouse models. *Cell Cycle* 17**,** 2019-2026.

Kent, L.W., Rahemtulla, F., and Michalek, S.M. (1999). Interleukin (IL)-1 and Porphyromonas gingivalis lipopolysaccharide stimulation of IL-6 production by fibroblasts derived from healthy or periodontally diseased human gingival tissue. *J Periodontol* 70**,** 274-282.

Kern, W.V., Engel, A., Schieffer, S., Prummer, O., and Kern, P. (1993). Circulating tumor necrosis factor alpha (TNF), soluble TNF receptors, and interleukin-6 in human subacute bacterial endocarditis. *Infect Immun* 61**,** 5413-5416.

Khan, K., Konar, M., Goyal, A., and Ghosh, S. (2010). Enteroaggregative Escherichia coli infection induces IL-8 production via activation of mitogen-activated protein kinases and the transcription factors NF-kappaB and AP-1 in INT-407 cells. *Mol Cell Biochem* 337**,** 17-24.

Khan, M.A., Bouzari, S., Ma, C., Rosenberger, C.M., Bergstrom, K.S., Gibson, D.L., Steiner, T.S., and Vallance, B.A. (2008). Flagellin-dependent and-independent inflammatory responses following infection by enteropathogenic Escherichia coli and Citrobacter rodentium. *Infection and immunity* 76**,** 1410-1422.

Khan, R., Basha, A., Goverdhanam, R., Rao, P.C., Tanemura, Y., Fujimoto, Y., and Begum, A.S. (2015). Attenuation of TNF-alpha secretion by L-proline-based cyclic dipeptides produced by culture broth of Pseudomonas aeruginosa. *Bioorg Med Chem Lett* 25**,** 5756-5761.

Kielian, T., Bearden, E.D., Baldwin, A.C., and Esen, N. (2004). IL-1 and TNF-alpha play a pivotal role in the host immune response in a mouse model of Staphylococcus aureus-induced experimental brain abscess. *J Neuropathol Exp Neurol* 63**,** 381-396.

Kim, J.R., Han, K., Han, Y., Kang, N., Shin, T.S., Park, H.J., Kim, H., Kwon, W., Lee, S., Kim, Y.K., Park, T., and Jang, J.Y. (2021). Microbiome Markers of Pancreatic Cancer Based on Bacteria-Derived Extracellular Vesicles Acquired from Blood Samples: A Retrospective Propensity Score Matching Analysis. *Biology (Basel)* 10.

Kim, N., Cho, S.I., Yim, J.Y., Kim, J.M., Lee, D.H., Park, J.H., Kim, J.S., Jung, H.C., and Song, I.S. (2006a). The effects of genetic polymorphisms of IL-1 and TNF-A on Helicobacter pylori-induced gastroduodenal diseases in Korea. *Helicobacter* 11**,** 105-112.

Kim, S.H., Lim, J.W., and Kim, H. (2018). Astaxanthin inhibits mitochondrial dysfunction and interleukin-8 expression in Helicobacter pylori-infected gastric epithelial cells. *Nutrients* 10**,** 1320.

Kim, S.O., Sheikh, H.I., Ha, S.D., Martins, A., and Reid, G. (2006b). G-CSF-mediated inhibition of JNK is a key mechanism for Lactobacillus rhamnosus-induced suppression of TNF production in macrophages. *Cell Microbiol* 8**,** 1958-1971.

Kimura, M., Matsukawa, A., Ohkawara, S., Takagi, K., and Yoshinaga, M. (1997). Blocking of TNF-alpha and IL-1 inhibits leukocyte infiltration at early, but not at late stage of S. aureus-induced arthritis and the concomitant cartilage destruction in rabbits. *Clin Immunol Immunopathol* 82**,** 18-25.

King, A.E., Schoenherr, E.M., Metten, M.R., and Dekrey, G.K. (2019). "Aryl hydrocarbon receptor activation does not impact the number of myeloid-derived suppressor cells in the spleens of mice immunized with cholera toxin". Am Assoc Immnol).

Kinoshita, S., Inoue, D., Sakuyama, T., Yoshizawa, A., Nagasaki, E., Arakawa, Y., Uwagawa, T., Kobayashi, K., Kobayashi, T., Saitoh, N., and Aiba, K. (2012). [A case of stage IV b pancreatic cancer in which a catheter-related infection caused by epidural/subcutaneous reservoir therapy affected palliative home-based care]. *Gan To Kagaku Ryoho* 39 Suppl 1**,** 61-63.

Kiyoi, T., Liu, S., and Mogi, M. (2022). "Effect of lysed Enterococcus faecalis FK-23 as a tyndallized probiotic in allergic diseases," in *Functional Foods and Nutraceuticals in Metabolic and Non-Communicable Diseases*. Elsevier), 459-461.

Knaus, U.G., Hertzberger, R., Pircalabioru, G.G., Yousefi, S.P., and Branco Dos Santos, F. (2017). Pathogen control at the intestinal mucosa - H2O2 to the rescue. *Gut Microbes* 8**,** 67-74.

Knuuti, J., Belevich, G., Sharma, V., Bloch, D.A., and Verkhovskaya, M. (2013). A single amino acid residue controls ROS production in the respiratory Complex I from Escherichia coli. *Mol Microbiol* 90**,** 1190-1200.

Korneev, K.V., Kondakova, A.N., Sviriaeva, E.N., Mitkin, N.A., Palmigiano, A., Kruglov, A.A., Telegin, G.B., Drutskaya, M.S., Sturiale, L., and Garozzo, D. (2018). Hypoacylated LPS from foodborne pathogen Campylobacter jejuni induces moderate TLR4-mediated inflammatory response in murine macrophages. *Frontiers in cellular and infection microbiology* 8**,** 58.

Kornmann, M., Kleeff, J., Debinski, W., and Korc, M. (1999). Pancreatic cancer cells express interleukin-13 and -4 receptors, and their growth is inhibited by Pseudomonas exotoxin coupled to interleukin-13 and -4. *Anticancer Res* 19**,** 125-131.

Kraft, M., Riedel, S., Maaser, C., Kucharzik, T., Steinbuechel, A., Domschke, W., and Luegering, N. (2001). IFN-gamma synergizes with TNF-alpha but not with viable H. pylori in up-regulating CXC chemokine secretion in gastric epithelial cells. *Clin Exp Immunol* 126**,** 474-481.

Kukita, A., Ichigi, Y., Takigawa, I., Watanabe, T., Kukita, T., and Miyamoto, H. (2012). Infection of RANKL-primed RAW-D macrophages with Porphyromonas gingivalis promotes osteoclastogenesis in a TNF-alpha-independent manner. *PLoS One* 7**,** e38500.

Kumar Pachathundikandi, S., Brandt, S., Madassery, J., and Backert, S. (2011). Induction of TLR-2 and TLR-5 expression by Helicobacter pylori switches cagPAI-dependent signalling leading to the secretion of IL-8 and TNF-alpha. *PLoS One* 6**,** e19614.

Kumar Pachathundikandi, S., Brandt, S., Madassery, J., and Backert, S. (2015). Correction: Induction of TLR-2 and TLR-5 Expression by Helicobacter pylori Switches cagPAI-Dependent Signalling Leading to the Secretion of IL-8 and TNF-alpha. *PLoS One* 10**,** e0141721.

Kumar, S., Metz, D.C., Kaplan, D.E., and Goldberg, D.S. (2020). The association of Helicobacter pylori with pancreatic cancer. *GastroHep* 2**,** 157-164.

Kunovsky, L., Dite, P., Jabandziev, P., Dolina, J., Vaculova, J., Blaho, M., Bojkova, M., Dvorackova, J., Uvirova, M., Kala, Z., and Trna, J. (2021). Helicobacter pylori infection and other bacteria in pancreatic cancer and autoimmune pancreatitis. *World J Gastrointest Oncol* 13**,** 835-844.

Kuo, S.H., Yeh, P.Y., Chen, L.T., Wu, M.S., Lin, C.W., Yeh, K.H., Tzeng, Y.S., Chen, J.Y., Hsu, P.N., Lin, J.T., and Cheng, A.L. (2008). Overexpression of B cell-activating factor of TNF family (BAFF) is associated with Helicobacter pylori-independent growth of gastric diffuse large B-cell lymphoma with histologic evidence of MALT lymphoma. *Blood* 112**,** 2927-2934.

Kuzuhara, T., Suganuma, M., Oka, K., and Fujiki, H. (2007). DNA-binding activity of TNF-alpha inducing protein from Helicobacter pylori. *Biochem Biophys Res Commun* 362**,** 805-810.

Kuzuhara, T., Suganuma, M., Tsuge, H., and Fujiki, H. (2005). Presence of a motif conserved between Helicobacter pylori TNF-alpha inducing protein (Tipalpha) and penicillin-binding proteins. *Biol Pharm Bull* 28**,** 2133-2137.

Lagoumintzis, G., Xaplanteri, P., Dimitracopoulos, G., and Paliogianni, F. (2008). TNF-alpha induction by Pseudomonas aeruginosa lipopolysaccharide or slime-glycolipoprotein in human monocytes is regulated at the level of Mitogen-activated Protein Kinase activity: a distinct role of Toll-like receptor 2 and 4. *Scand J Immunol* 67**,** 193-203.

Laya, G.B., Anandhi, A., Gurushankari, B., Mandal, J., and Kate, V. (2022). Association Between Helicobacter pylori and Periampullary and Pancreatic Cancer: a Case-Control Study. *J Gastrointest Cancer*.

Le, X.K., Laflamme, C., and Rouabhia, M. (2009). Porphyromonas gingivalis decreases osteoblast proliferation through IL-6-RANKL/OPG and MMP-9/TIMPs pathways. *Indian J Dent Res* 20**,** 141-149.

Leber, A., Hontecillas, R., Zoccoli-Rodriguez, V., Chauhan, J., and Bassaganya-Riera, J. (2019). Oral Treatment with BT-11 Ameliorates Inflammatory Bowel Disease by Enhancing Regulatory T Cell Responses in the Gut. *J Immunol* 202**,** 2095-2104.

Lee, C.W., Wu, C.H., Chiang, Y.C., Chen, Y.L., Chang, K.T., Chuang, C.C., and Lee, I.T. (2018a). Carbon monoxide releasing molecule-2 attenuates Pseudomonas aeruginosa-induced ROS-dependent ICAM-1 expression in human pulmonary alveolar epithelial cells. *Redox Biol* 18**,** 93-103.

Lee, J., Yoon, Y.J., Kim, J.H., Dinh, N.T.H., Go, G., Tae, S., Park, K.S., Park, H.T., Lee, C., Roh, T.Y., Di Vizio, D., and Gho, Y.S. (2018b). Outer Membrane Vesicles Derived From Escherichia coli Regulate Neutrophil Migration by Induction of Endothelial IL-8. *Front Microbiol* 9**,** 2268.

Lee, K., Oh, H.J., Kang, M.S., Kim, S., Ahn, S., Kim, M.J., Kim, S.W., and Chang, S. (2021). Metagenomic analysis of gut microbiome reveals a dynamic change in Alistipes onderdonkii in the preclinical model of pancreatic cancer, suppressing its proliferation. *Appl Microbiol Biotechnol* 105**,** 8343-8358.

Lee, M.K., Lee, Y., Huh, J.W., Chen, H., Wu, W., and Ha, U.H. (2020). The Pseudomonas aeruginosa HSP90-like protein HtpG regulates IL-8 expression through NF-kappaB/p38 MAPK and CYLD signaling triggered by TLR4 and CD91. *Microbes Infect* 22**,** 558-566.

Léger, L., Budin-Verneuil, A., Cacaci, M., Benachour, A., Hartke, A., and Verneuil, N. (2019). β-lactam exposure triggers reactive oxygen species formation in enterococcus faecalis via the respiratory chain component DMK. *Cell Reports* 29**,** 2184-2191. e2183.

Leidal, K.G., Munson, K.L., and Denning, G.M. (2001). Small molecular weight secretory factors from Pseudomonas aeruginosa have opposite effects on IL-8 and RANTES expression by human airway epithelial cells. *Am J Respir Cell Mol Biol* 25**,** 186-195.

Leschner, S., Westphal, K., Dietrich, N., Viegas, N., Jablonska, J., Lyszkiewicz, M., Lienenklaus, S., Falk, W., Gekara, N., Loessner, H., and Weiss, S. (2009). Tumor invasion of Salmonella enterica serovar Typhimurium is accompanied by strong hemorrhage promoted by TNF-alpha. *PLoS One* 4**,** e6692.

Lesiow, M.K., Bienko, A., Sobanska, K., Kowalik-Jankowska, T., Rolka, K., Legowska, A., and Ptaszynska, N. (2020). Cu(II) complexes with peptides from FomA protein containing -His-Xaa-Yaa-Zaa-His and -His-His-motifs. ROS generation and DNA degradation. *J Inorg Biochem* 212**,** 111250.

Lesiow, M.K., Komarnicka, U.K., Kyziol, A., Bienko, A., and Pietrzyk, P. (2019). ROS-mediated lipid peroxidation as a result of Cu(ii) interaction with FomA protein fragments of F. nucleatum: relevance to colorectal carcinogenesis. *Metallomics* 11**,** 2066-2077.

Lesiow, M.K., Komarnicka, U.K., Stokowa-Soltys, K., Rolka, K., Legowska, A., Ptaszynska, N., Wieczorek, R., Kyziol, A., and Jezowska-Bojczuk, M. (2018). Relationship between copper(ii) complexes with FomA adhesin fragments of F. nucleatum and colorectal cancer. Coordination pattern and ability to promote ROS production. *Dalton Trans* 47**,** 5445-5458.

Leung, Y.H., Ng, A.M., Xu, X., Shen, Z., Gethings, L.A., Wong, M.T., Chan, C.M., Guo, M.Y., Ng, Y.H., Djurisic, A.B., Lee, P.K., Chan, W.K., Yu, L.H., Phillips, D.L., Ma, A.P., and Leung, F.C. (2014). Mechanisms of antibacterial activity of MgO: non-ROS mediated toxicity of MgO nanoparticles towards Escherichia coli. *Small* 10**,** 1171-1183.

Li, B., Li, J., Pan, X., Ding, G., Cao, H., Jiang, W., Zheng, J., and Zhou, H. (2010). Artesunate protects sepsis model mice challenged with Staphylococcus aureus by decreasing TNF-alpha release via inhibition TLR2 and Nod2 mRNA expressions and transcription factor NF-kappaB activation. *Int Immunopharmacol* 10**,** 344-350.

Li, C., Xia, B., Yang, Y., Li, J., and Xia, H.H. (2005). TNF gene polymorphisms and Helicobacter Pylori infection in gastric carcinogenesis in Chinese population. *Am J Gastroenterol* 100**,** 290-294.

Li, D., Duell, E.J., Yu, K., Risch, H.A., Olson, S.H., Kooperberg, C., Wolpin, B.M., Jiao, L., Dong, X., Wheeler, B., Arslan, A.A., Bueno-De-Mesquita, H.B., Fuchs, C.S., Gallinger, S., Gross, M., Hartge, P., Hoover, R.N., Holly, E.A., Jacobs, E.J., Klein, A.P., Lacroix, A., Mandelson, M.T., Petersen, G., Zheng, W., Agalliu, I., Albanes, D., Boutron-Ruault, M.C., Bracci, P.M., Buring, J.E., Canzian, F., Chang, K., Chanock, S.J., Cotterchio, M., Gaziano, J.M., Giovannucci, E.L., Goggins, M., Hallmans, G., Hankinson, S.E., Hoffman Bolton, J.A., Hunter, D.J., Hutchinson, A., Jacobs, K.B., Jenab, M., Khaw, K.T., Kraft, P., Krogh, V., Kurtz, R.C., Mcwilliams, R.R., Mendelsohn, J.B., Patel, A.V., Rabe, K.G., Riboli, E., Shu, X.O., Tjonneland, A., Tobias, G.S., Trichopoulos, D., Virtamo, J., Visvanathan, K., Watters, J., Yu, H., Zeleniuch-Jacquotte, A., Amundadottir, L., and Stolzenberg-Solomon, R.Z. (2012). Pathway analysis of genome-wide association study data highlights pancreatic development genes as susceptibility factors for pancreatic cancer. *Carcinogenesis* 33**,** 1384-1390.

Li, H., Luo, Y.F., Wang, Y.S., Yang, Q., Xiao, Y.L., Cai, H.R., and Xie, C.M. (2018). Using ROS as a Second Messenger, NADPH Oxidase 2 Mediates Macrophage Senescence via Interaction with NF-kappaB during Pseudomonas aeruginosa Infection. *Oxid Med Cell Longev* 2018**,** 9741838.

Li, J., Peccerillo, J., Kaley, K., and Saif, M.W. (2009). Staphylococcus aureus bacteremia related with erlotinib skin toxicity in a patient with pancreatic cancer. *JOP* 10**,** 338-340.

Li, M., Wei, L., Zhou, W., He, Z., Ran, S., and Liang, J. (2020a). miR-200a contributes to the migration of BMSCs induced by the secretions of E. faecalis via FOXJ1/NFκB/MMPs axis. *Stem Cell Research & Therapy* 11**,** 1-12.

Li, P., Shu, Y., and Gu, Y. (2020b). The potential role of bacteria in pancreatic cancer: a systematic review. *Carcinogenesis* 41**,** 397-404.

Li, W., Zhou, X., Cai, J., Zhao, F., Cao, T., Ning, L., Luo, C., Xiao, X., and Liu, S. (2021). Recombinant Treponema pallidum protein Tp0768 promotes proinflammatory cytokine secretion of macrophages through ER stress and ROS/NF-kappaB pathway. *Appl Microbiol Biotechnol* 105**,** 353-366.

Li, X., Liu, S., Luo, J., Liu, A., Tang, S., Liu, S., Yu, M., and Zhang, Y. (2015). Helicobacter pylori induces IL-1beta and IL-18 production in human monocytic cell line through activation of NLRP3 inflammasome via ROS signaling pathway. *Pathog Dis* 73.

Li, Y., Niu, J., Zhang, W., Zhang, L., and Shang, E. (2014). Influence of aqueous media on the ROS-mediated toxicity of ZnO nanoparticles toward green fluorescent protein-expressing Escherichia coli under UV-365 irradiation. *Langmuir* 30**,** 2852-2862.

Li, Z., Fallon, J., Mandeli, J., Wetmur, J., and Woo, S.L. (2008). A genetically enhanced anaerobic bacterium for oncopathic therapy of pancreatic cancer. *J Natl Cancer Inst* 100**,** 1389-1400.

Lian, D., Dai, L., Xie, Z., Zhou, X., Liu, X., Zhang, Y., Huang, Y., and Chen, Y. (2018). Periodontal ligament fibroblasts migration injury via ROS/TXNIP/Nlrp3 inflammasome pathway with Porphyromonas gingivalis lipopolysaccharide. *Mol Immunol* 103**,** 209-219.

Liang, G., Wang, H., Shi, H., Zhu, M., An, J., Qi, Y., Du, J., Li, Y., and Gao, S. (2020). Porphyromonas gingivalis promotes the proliferation and migration of esophageal squamous cell carcinoma through the miR-194/GRHL3/PTEN/Akt axis. *ACS Infectious Diseases* 6**,** 871-881.

Liang, X., and Ji, Y. (2007). Involvement of alpha5beta1-integrin and TNF-alpha in Staphylococcus aureus alpha-toxin-induced death of epithelial cells. *Cell Microbiol* 9**,** 1809-1821.

Lin, F., Meng, X., Guo, Y., Cao, W., Liu, W., Xia, Q., Hui, Z., Chen, J., Hong, S., Zhang, X., Wu, C., Wang, D., Wang, J., Lu, L., Qian, W., Wei, L., and Wang, L. (2019). Epigenetic initiation of the TH17 differentiation program is promoted by Cxxc finger protein 1. *Sci Adv* 5**,** eaax1608.

Lin, T.Y., Lan, W.H., Chiu, Y.F., Feng, C.L., Chiu, C.H., Kuo, C.J., and Lai, C.H. (2021). Statins' Regulation of the Virulence Factors of Helicobacter pylori and the Production of ROS May Inhibit the Development of Gastric Cancer. *Antioxidants (Basel)* 10.

Lin, W.C., Tsai, H.F., Liao, H.J., Tang, C.H., Wu, Y.Y., Hsu, P.I., Cheng, A.L., and Hsu, P.N. (2014). Helicobacter pylori sensitizes TNF-related apoptosis-inducing ligand (TRAIL)-mediated apoptosis in human gastric epithelial cells through regulation of FLIP. *Cell Death Dis* 5**,** e1109.

Lindkvist, B., Johansen, D., Borgstrom, A., and Manjer, J. (2008). A prospective study of Helicobacter pylori in relation to the risk for pancreatic cancer. *BMC Cancer* 8**,** 321.

Linevsky, J.K., Pothoulakis, C., Keates, S., Warny, M., Keates, A.C., Lamont, J.T., and Kelly, C.P. (1997). IL-8 release and neutrophil activation by Clostridium difficile toxin-exposed human monocytes. *Am J Physiol* 273**,** G1333-1340.

Liquete, E., Ali, S., Kammo, R., Ali, M., Alali, F., Challa, H., and Fata, F. (2012). Acute Generalized Exanthematous Pustulosis Induced by Erlotinib (Tarceva) with Superimposed Staphylococcus aureus Skin Infection in a Pancreatic Cancer Patient: A Case Report. *Case Rep Oncol* 5**,** 253-259.

Liu, H., Chen, Y.T., Wang, R., and Chen, X.Z. (2017). Helicobacter pylori infection, atrophic gastritis, and pancreatic cancer risk: A meta-analysis of prospective epidemiologic studies. *Medicine (Baltimore)* 96**,** e7811.

Liu, J., Chen, X., Zhou, J., Ye, L., Yang, D., and Song, Y. (2020). Particulate matter exposure promotes Pseudomonas aeruginosa invasion into airway epithelia by upregulating PAFR via the ROS-mediated PI3K pathway. *Hum Cell* 33**,** 963-973.

Liu, J., Song, N., Huang, Y., and Chen, Y. (2018). Irisin inhibits pancreatic cancer cell growth via the AMPK-mTOR pathway. *Sci Rep* 8**,** 15247.

Liu, J., Wang, Y., and Ouyang, X. (2014). Beyond toll-like receptors: Porphyromonas gingivalis induces IL-6, IL-8, and VCAM-1 expression through NOD-mediated NF-kappaB and ERK signaling pathways in periodontal fibroblasts. *Inflammation* 37**,** 522-533.

Liu, T., and Chopra, A.K. (2010). An enteric pathogen Salmonella enterica serovar Typhimurium suppresses tumor growth by downregulating CD44high and CD4T regulatory (Treg) cell expression in mice: the critical role of lipopolysaccharide and Braun lipoprotein in modulating tumor growth. *Cancer Gene Ther* 17**,** 97-108.

Liu, Z.Z., Chen, W., Zhou, C.K., Ma, K., Gao, Y., and Yang, Y.J. (2022). Stimulator of Interferon Genes (STING) Promotes Staphylococcus aureus-Induced Extracellular Traps Formation via the ROS-ERK Signaling Pathway. *Front Cell Dev Biol* 10**,** 836880.

Long, X., Li, X., Li, T., Yan, Q., Wen, L., Yang, X., Li, H., and Sun, L. (2021). Umbilical cord mesenchymal stem cells enhance the therapeutic effect of imipenem by regulating myeloid-derived suppressor cells in septic mice. *Annals of Translational Medicine* 9.

Lopez-Colom, P., Yu, K., Barba-Vidal, E., Saco, Y., Martin-Orue, S.M., Castillejos, L., Sola-Oriol, D., and Bassols, A. (2019). I-FABP, Pig-MAP and TNF-alpha as biomarkers for monitoring gut-wall integrity in front of Salmonella Typhimurium and ETEC K88 infection in a weaned piglet model. *Res Vet Sci* 124**,** 426-432.

Lowenfels, A.B., and Maisonneuve, P. (2002). Epidemiologic and etiologic factors of pancreatic cancer. *Hematol Oncol Clin North Am* 16**,** 1-16.

Lu, C.C., Sheu, B.S., Chen, T.W., Yang, H.B., Hung, K.H., Kao, A.W., Chuang, C.H., and Wu, J.J. (2005). Host TNF-alpha-1031 and -863 promoter single nucleotide polymorphisms determine the risk of benign ulceration after H. pylori infection. *Am J Gastroenterol* 100**,** 1274-1282.

Lu, H., Han, X., Ren, J., Ren, K., Li, Z., and Zhang, Q. (2021). Metformin attenuates synergic effect of diabetes mellitus and Helicobacter pylori infection on gastric cancer cells proliferation by suppressing PTEN expression. *Journal of Cellular and Molecular Medicine* 25**,** 4534-4542.

Lu, J., Ma, S.S., Zhang, W.Y., and Duan, J.P. (2019). Changes in peripheral blood inflammatory factors (TNF-alpha and IL-6) and intestinal flora in AIDS and HIV-positive individuals. *J Zhejiang Univ Sci B* 20**,** 793-802.

Lu, S., Wang, J., He, Z., He, S., Zheng, K., Xu, M., Yuan, S., and Wu, Y. (2022). Treponema pallidum Tp0751 alters the expression of tight junction proteins by promoting bEnd3 cell apoptosis and IL-6 secretion. *Int J Med Microbiol* 312**,** 151553.

Lu, Y., Rong, J., Lai, Y., Tao, L., Yuan, X., and Shu, X. (2020). The Degree of Helicobacter pylori Infection Affects the State of Macrophage Polarization through Crosstalk between ROS and HIF-1alpha. *Oxid Med Cell Longev* 2020**,** 5281795.

Luo, J., Nordenvall, C., Nyren, O., Adami, H.O., Permert, J., and Ye, W. (2007). The risk of pancreatic cancer in patients with gastric or duodenal ulcer disease. *Int J Cancer* 120**,** 368-372.

Luo, W., Cao, Z., Qiu, J., Liu, Y., Zheng, L., and Zhang, T. (2020). Novel Discoveries Targeting Pathogenic Gut Microbes and New Therapies in Pancreatic Cancer: Does Pathogenic E. coli Infection Cause Pancreatic Cancer Progression Modulated by TUBB/Rho/ROCK Signaling Pathway? A Bioinformatic Analysis. *Biomed Res Int* 2020**,** 2340124.

Luo, W., Wang, C.Y., and Jin, L. (2012). Baicalin downregulates Porphyromonas gingivalis lipopolysaccharide-upregulated IL-6 and IL-8 expression in human oral keratinocytes by negative regulation of TLR signaling. *PLoS One* 7**,** e51008.

Luo, X., Zhang, X., Gan, L., Zhou, C., Zhao, T., Zeng, T., Liu, S., Xiao, Y., Yu, J., and Zhao, F. (2018). The outer membrane protein Tp92 of Treponema pallidum induces human mononuclear cell death and IL-8 secretion. *J Cell Mol Med* 22**,** 6039-6054.

Lv, C., Yang, S., Chen, X., Zhu, X., Lin, W., Wang, L., Huang, Z., Wang, M., and Tu, G. (2017). MicroRNA-21 promotes bone mesenchymal stem cells migration in vitro by activating PI3K/Akt/MMPs pathway. *Journal of Clinical Neuroscience* 46**,** 156-162.

Maisey, H.C., Doran, K.S., and Nizet, V. (2008). Recent advances in understanding the molecular basis of group B Streptococcus virulence. *Expert reviews in molecular medicine* 10.

Maisonneuve, P., and Lowenfels, A.B. (2010). Epidemiology of pancreatic cancer: an update. *Dig Dis* 28**,** 645-656.

Maisonneuve, P., and Lowenfels, A.B. (2015). Risk factors for pancreatic cancer: a summary review of meta-analytical studies. *Int J Epidemiol* 44**,** 186-198.

Matsumoto, T., Kawakami, Y., Oana, K., Honda, T., Yamauchi, K., Okimura, Y., Shiohara, M., and Kasuga, E. (2006). First isolation of Dysgonomonas mossii from intestinal juice of a patient with pancreatic cancer. *Arch Med Res* 37**,** 914-916.

Matuschak, G.M., Munoz, C., Epperly, N.A., Britton, R.S., Walsh, D., Schilly, D.R., Tredway, T.L., Khan, T.A., Bacon, B.R., and Lechner, A.J. (1994). TNF-alpha and IL-6 expression in perfused rat liver after intraportal candidemia vs. E. coli or S. aureus bacteremia. *Am J Physiol* 267**,** R446-454.

Mcdew-White, M., Lee, E., Alvarez, X., Sestak, K., Ling, B.J., Byrareddy, S.N., Okeoma, C.M., and Mohan, M. (2022). Cannabinoid control of gingival immune activation in chronically SIV-infected rhesus macaques involves modulation of the indoleamine-2,3-dioxygenase-1 pathway and salivary microbiome. *EBioMedicine* 75**,** 103769.

Mei, Q.X., Huang, C.L., Luo, S.Z., Zhang, X.M., Zeng, Y., and Lu, Y.Y. (2018). Characterization of the duodenal bacterial microbiota in patients with pancreatic head cancer vs. healthy controls. *Pancreatology* 18**,** 438-445.

Melo Barbosa, H.P., Martins, L.C., Dos Santos, S.E., Demachki, S., Assumpcao, M.B., Aragao, C.D., and De Oliveira Corvelo, T.C. (2009). Interleukin-1 and TNF-alpha polymorphisms and Helicobacter pylori in a Brazilian Amazon population. *World J Gastroenterol* 15**,** 1465-1471.

Meraz, I.M., Arikawa, K., Nakamura, H., Ogasawara, J., Hase, A., and Nishikawa, Y. (2007). Association of IL-8-inducing strains of diffusely adherent Escherichia coli with sporadic diarrheal patients with less than 5 years of age. *Braz J Infect Dis* 11**,** 44-49.

Merlin, T., Gumenscheimer, M., Galanos, C., and Freudenberg, M.A. (2001). TNF-alpha hyper-responses to Gram-negative and Gram-positive bacteria in Propionibacterium acnes primed or Salmonella typhimurium infected mice. *J Endotoxin Res* 7**,** 157-163.

Michaud, D.S. (2013). Role of bacterial infections in pancreatic cancer. *Carcinogenesis* 34**,** 2193-2197.

Michaud, D.S., Izard, J., Wilhelm-Benartzi, C.S., You, D.H., Grote, V.A., Tjonneland, A., Dahm, C.C., Overvad, K., Jenab, M., Fedirko, V., Boutron-Ruault, M.C., Clavel-Chapelon, F., Racine, A., Kaaks, R., Boeing, H., Foerster, J., Trichopoulou, A., Lagiou, P., Trichopoulos, D., Sacerdote, C., Sieri, S., Palli, D., Tumino, R., Panico, S., Siersema, P.D., Peeters, P.H., Lund, E., Barricarte, A., Huerta, J.M., Molina-Montes, E., Dorronsoro, M., Quiros, J.R., Duell, E.J., Ye, W., Sund, M., Lindkvist, B., Johansen, D., Khaw, K.T., Wareham, N., Travis, R.C., Vineis, P., Bueno-De-Mesquita, H.B., and Riboli, E. (2013). Plasma antibodies to oral bacteria and risk of pancreatic cancer in a large European prospective cohort study. *Gut* 62**,** 1764-1770.

Mitchell, J., Kim, S.J., Koukos, G., Seelmann, A., Veit, B., Shepard, B., Blumer-Schuette, S., Winter, H.S., Iliopoulos, D., Pothoulakis, C., Im, E., and Rhee, S.H. (2018). Colonic Inhibition of Phosphatase and Tensin Homolog Increases Colitogenic Bacteria, Causing Development of Colitis in Il10-/- Mice. *Inflamm Bowel Dis* 24**,** 1718-1732.

Mitsui-Yamaguchi, T., Abe, A., Danbara, H., and Kawahara, K. (1997). Induction of TNF-alpha mRNA in murine macrophages by virulent and avirulent strains of Salmonella choleraesuis serovar typhimurium and serovar Choleraesuis. *Microb Pathog* 22**,** 59-66.

Miyamoto, M., Ishihara, K., and Okuda, K. (2006). The Treponema denticola surface protease dentilisin degrades interleukin-1 beta (IL-1 beta), IL-6, and tumor necrosis factor alpha. *Infect Immun* 74**,** 2462-2467.

Moayedi, A., Nowroozi, J., and Akhavan Sepahy, A. (2018). Cytotoxic effect of pyocyanin on human pancreatic cancer cell line (Panc-1). *Iran J Basic Med Sci* 21**,** 794-799.

Mohammed, H., Varoni, E.M., Cochis, A., Cordaro, M., Gallenzi, P., Patini, R., Staderini, E., Lajolo, C., Rimondini, L., and Rocchetti, V. (2018). Oral Dysbiosis in Pancreatic Cancer and Liver Cirrhosis: A Review of the Literature. *Biomedicines* 6.

Mohinta, S., Kannan, A.K., Gowda, K., Amin, S.G., Perdew, G.H., and August, A. (2015). Differential regulation of Th17 and T regulatory cell differentiation by aryl hydrocarbon receptor dependent xenobiotic response element dependent and independent pathways. *Toxicol Sci* 145**,** 233-243.

Monick, M.M., Powers, L.S., Butler, N.S., and Hunninghake, G.W. (2003). Inhibition of Rho family GTPases results in increased TNF-alpha production after lipopolysaccharide exposure. *J Immunol* 171**,** 2625-2630.

Moradipour, A., Khosravi, A., and Piri, F. (2018). Fecal Helicobacter pylori glmM and 16S rRNA genes correlate with serum TNF-alpha and IL-1beta cytokine fluctuations. *Acta Microbiol Immunol Hung* 65**,** 489-499.

Morales, E.H., Collao, B., Desai, P.T., Calderon, I.L., Gil, F., Luraschi, R., Porwollik, S., Mcclelland, M., and Saavedra, C.P. (2013). Probing the ArcA regulon under aerobic/ROS conditions in Salmonella enterica serovar Typhimurium. *BMC Genomics* 14**,** 626.

Morandini, A.C., Sipert, C.R., Gasparoto, T.H., Greghi, S.L., Passanezi, E., Rezende, M.L., Sant'ana, A.P., Campanelli, A.P., Garlet, G.P., and Santos, C.F. (2010). Differential production of macrophage inflammatory protein-1alpha, stromal-derived factor-1, and IL-6 by human cultured periodontal ligament and gingival fibroblasts challenged with lipopolysaccharide from P. gingivalis. *J Periodontol* 81**,** 310-317.

Morgell, A., Reisz, J.A., Ateeb, Z., Davanian, H., Reinsbach, S.E., Halimi, A., Gaiser, R., Valente, R., Arnelo, U., Chiaro, M.D., Sallberg Chen, M., and D'alessandro, A. (2020). Metabolic characterization of plasma and cyst fluid from cystic precursors to pancreatic cancer patients reveal metabolic signatures of bacterial infection. *medRxiv*.

Morgell, A., Reisz, J.A., Ateeb, Z., Davanian, H., Reinsbach, S.E., Halimi, A., Gaiser, R., Valente, R., Arnelo, U., Del Chiaro, M., Chen, M.S., and D'alessandro, A. (2021). Metabolic Characterization of Plasma and Cyst Fluid from Cystic Precursors to Pancreatic Cancer Patients Reveal Metabolic Signatures of Bacterial Infection. *J Proteome Res* 20**,** 2725-2738.

Morningstar-Wright, L., Czinn, S.J., Piazuelo, M.B., Banerjee, A., Godlewska, R., and Blanchard, T.G. (2022). The TNF-Alpha Inducing Protein is Associated With Gastric Inflammation and Hyperplasia in a Murine Model of Helicobacter pylori Infection. *Front Pharmacol* 13**,** 817237.

Murakami, T., Hiroshima, Y., Zhang, Y., Zhao, M., Kiyuna, T., Hwang, H.K., Miyake, K., Homma, Y., Mori, R., Matsuyama, R., Chishima, T., Ichikawa, Y., Tanaka, K., Bouvet, M., Endo, I., and Hoffman, R.M. (2018). Tumor-Targeting Salmonella typhimurium A1-R Promotes Tumoricidal CD8(+) T Cell Tumor Infiltration and Arrests Growth and Metastasis in a Syngeneic Pancreatic-Cancer Orthotopic Mouse Model. *J Cell Biochem* 119**,** 634-639.

Murakami, Y., Hanazawa, S., Nishida, K., Iwasaka, H., and Kitano, S. (1993). N-acetyl-D-galactosamine inhibits TNF-alpha gene expression induced in mouse peritoneal macrophages by fimbriae of Porphyromonas (Bacteroides) gingivalis, an oral anaerobe. *Biochem Biophys Res Commun* 192**,** 826-832.

Nagakura, C., Hayashi, K., Zhao, M., Yamauchi, K., Yamamoto, N., Tsuchiya, H., Tomita, K., Bouvet, M., and Hoffman, R.M. (2009). Efficacy of a genetically-modified Salmonella typhimurium in an orthotopic human pancreatic cancer in nude mice. *Anticancer Res* 29**,** 1873-1878.

Nakahashi-Oda, C., Udayanga, K.G., Nakamura, Y., Nakazawa, Y., Totsuka, N., Miki, H., Iino, S., Tahara-Hanaoka, S., Honda, S., Shibuya, K., and Shibuya, A. (2016). Apoptotic epithelial cells control the abundance of Treg cells at barrier surfaces. *Nat Immunol* 17**,** 441-450.

Nakamoto, K., Watanabe, M., Sada, M., Inui, T., Nakamura, M., Honda, K., Wada, H., Ishii, H., and Takizawa, H. (2019). Pseudomonas aeruginosa-derived flagellin stimulates IL-6 and IL-8 production in human bronchial epithelial cells: A potential mechanism for progression and exacerbation of COPD. *Exp Lung Res* 45**,** 255-266.

Nakstad, B., Sonerud, T., and Solevag, A.L. (2016). Early detection of neonatal group B streptococcus sepsis and the possible diagnostic utility of IL-6, IL-8, and CD11b in a human umbilical cord blood in vitro model. *Infect Drug Resist* 9**,** 171-179.

Nandi, A., and Bishayi, B. (2017). A novel CCR-2/TLR-2 triggered signaling in murine peritoneal macrophages intensifies bacterial (Staphylococcus aureus) killing by reactive oxygen species through TNF-R1. *Immunol Lett* 190**,** 93-107.

Nebel, D., Arvidsson, J., Lillqvist, J., Holm, A., and Nilsson, B.O. (2013). Differential effects of LPS from Escherichia coli and Porphyromonas gingivalis on IL-6 production in human periodontal ligament cells. *Acta Odontol Scand* 71**,** 892-898.

Negovan, A., Iancu, M., Tripon, F., Crauciuc, A., Mocan, S., and Banescu, C. (2021). Cytokine TGF-beta1, TNF-alpha, IFN-gamma and IL-6 Gene Polymorphisms and Localization of Premalignant Gastric Lesions in Immunohistochemically H. pylori-negative Patients. *Int J Med Sci* 18**,** 2743-2751.

Ng, E.K., Panesar, N., Longo, W.E., Shapiro, M.J., Kaminski, D.L., Tolman, K.C., and Mazuski, J.E. (2003). Human intestinal epithelial and smooth muscle cells are potent producers of IL-6. *Mediators Inflamm* 12**,** 3-8.

Ngo, H.K., Lee, H.G., Piao, J.Y., Zhong, X., Lee, H.N., Han, H.J., Kim, W., Kim, D.H., Cha, Y.N., Na, H.K., and Surh, Y.J. (2016). Helicobacter pylori induces Snail expression through ROS-mediated activation of Erk and inactivation of GSK-3beta in human gastric cancer cells. *Mol Carcinog* 55**,** 2236-2246.

Nilsson, H.O., Stenram, U., Ihse, I., and Wadstrom, T. (2002). Re: Helicobacter pylori seropositivity as a risk factor for pancreatic cancer. *J Natl Cancer Inst* 94**,** 632-633.

Nishimura, J., Sato, K., Sugamori, T., Muranaka, H., Tomoshima, Y., Takahashi, T., Okamoto, T., and Ichian, H. (1998). [Effects of erythromycin on Pseudomonas aeruginosa pyocyanine-induced IL-8 production and its biological activity (the 2nd report)]. *Jpn J Antibiot* 51 Suppl A**,** 116-119.

Noster, J., Persicke, M., Chao, T.C., Krone, L., Heppner, B., Hensel, M., and Hansmeier, N. (2019). Impact of ROS-Induced Damage of TCA Cycle Enzymes on Metabolism and Virulence of Salmonella enterica serovar Typhimurium. *Front Microbiol* 10**,** 762.

Nymo, S., Gustavsen, A., Nilsson, P.H., Lau, C., Espevik, T., and Mollnes, T.E. (2016). Human Endothelial Cell Activation by Escherichia coli and Staphylococcus aureus Is Mediated by TNF and IL-1beta Secondarily to Activation of C5 and CD14 in Whole Blood. *J Immunol* 196**,** 2293-2299.

O'brien, G.J., Riddell, G., Elborn, J.S., Ennis, M., and Skibinski, G. (2006). Staphylococcus aureus enterotoxins induce IL-8 secretion by human nasal epithelial cells. *Respir Res* 7**,** 115.

Ogawa, T., and Uchida, H. (1996). Differential induction of IL-1 beta and IL-6 production by the nontoxic lipid A from Porphyromonas gingivalis in comparison with synthetic Escherichia coli lipid A in human peripheral blood mononuclear cells. *FEMS Immunol Med Microbiol* 14**,** 1-13.

Ogrendik, M. (2015). Oral bacteria in pancreatic cancer: mutagenesis of the p53 tumour suppressor gene. *Int J Clin Exp Pathol* 8**,** 11835-11836.

Ogrendik, M. (2017). Periodontal Pathogens in the Etiology of Pancreatic Cancer. *Gastrointest Tumors* 3**,** 125-127.

Ogura, K., Terasaki, Y., Miyoshi-Akiyama, T., Terasaki, M., Moss, J., Noda, M., and Yahiro, K. (2017). Vibrio cholerae Cholix Toxin-Induced HepG2 Cell Death is Enhanced by Tumor Necrosis Factor-Alpha Through ROS and Intracellular Signal-Regulated Kinases. *Toxicol Sci* 156**,** 455-468.

Okutomi, T., Ubukata, T., Yamaoka, K., Abe, S., and Yamaguchi, H. (1997). Augmentation of production of TNF-alpha and anti-tumour activity by an amphotericin B preparation for clinical use in mice. *Br J Cancer* 75**,** 1613-1616.

Osawa, M. (2018). A repetitive mutation and selection system for bacterial evolution to increase the specific affinity to pancreatic cancer cells. *PLoS One* 13**,** e0198157.

Oviedo-Boyso, J., Barriga-Rivera, J.G., Valdez-Alarcon, J.J., Bravo-Patino, A., Carabez-Trejo, A., Cajero-Juarez, M., and Baizabal-Aguirre, V.M. (2008a). Internalization of Staphylococcus aureus by bovine endothelial cells is associated with the activity state of NF-kappaB and modulated by the pro-inflammatory cytokines TNF-alpha and IL-1beta. *Scand J Immunol* 67**,** 169-176.

Oviedo-Boyso, J., Cardoso-Correa, B.I., Cajero-Juarez, M., Bravo-Patino, A., Valdez-Alarcon, J.J., and Baizabal-Aguirre, V.M. (2008b). The capacity of bovine endothelial cells to eliminate intracellular Staphylococcus aureus and Staphylococcus epidermidis is increased by the proinflammatory cytokines TNF-alpha and IL-1beta. *FEMS Immunol Med Microbiol* 54**,** 53-59.

Panebianco, C., Adamberg, K., Jaagura, M., Copetti, M., Fontana, A., Adamberg, S., Kolk, K., Vilu, R., Andriulli, A., and Pazienza, V. (2018). Influence of gemcitabine chemotherapy on the microbiota of pancreatic cancer xenografted mice. *Cancer Chemother Pharmacol* 81**,** 773-782.

Panebianco, C., and Pazienza, V. (2019). Body site-dependent variations of microbiota in pancreatic cancer pathophysiology. *Crit Rev Clin Lab Sci* 56**,** 260-273.

Pathak, S.K., Tavares, R., De Klerk, N., Spetz, A.L., and Jonsson, A.B. (2013). Helicobacter pylori protein JHP0290 binds to multiple cell types and induces macrophage apoptosis via tumor necrosis factor (TNF)-dependent and independent pathways. *PLoS One* 8**,** e77872.

Permuth, J.B., Rahman, S., Chen, D.T., Waterboer, T., and Giuliano, A.R. (2021). A Case Control Study of the Seroprevalence of Helicobacter pylori Proteins and Their Association with Pancreatic Cancer Risk. *J Pancreat Cancer* 7**,** 57-64.

Petelin, M., Naruishi, K., Shiomi, N., Mineshiba, J., Arai, H., Nishimura, F., Takashiba, S., and Murayama, Y. (2004). Systemic up-regulation of sTNFR2 and IL-6 in Porphyromonas gingivalis pneumonia in mice. *Exp Mol Pathol* 76**,** 76-81.

Petrick, J.L., Wilkinson, J.E., Michaud, D.S., Cai, Q., Gerlovin, H., Signorello, L.B., Wolpin, B.M., Ruiz-Narvaez, E.A., Long, J., Yang, Y., Johnson, W.E., Shu, X.O., Huttenhower, C., and Palmer, J.R. (2022). The oral microbiome in relation to pancreatic cancer risk in African Americans. *Br J Cancer* 126**,** 287-296.

Polak, D., Yaya, A., Levy, D.H., Metzger, Z., and Abramovitz, I. (2021). Enterococcus faecalis sustained infection induces macrophage pro‐resolution polarization. *International Endodontic Journal* 54**,** 1840-1849.

Poysti, S., Silojarvi, S., Toivonen, R., and Hanninen, A. (2021). Plasmacytoid dendritic cells regulate host immune response to Citrobacter rodentium induced colitis in colon-draining lymph nodes. *Eur J Immunol* 51**,** 620-625.

Pozzobon, T., Facchinello, N., Bossi, F., Capitani, N., Benagiano, M., Di Benedetto, G., Zennaro, C., West, N., Codolo, G., Bernardini, M., Baldari, C.T., D'elios, M.M., Pellegrini, L., Argenton, F., and De Bernard, M. (2016). Treponema pallidum (syphilis) antigen TpF1 induces angiogenesis through the activation of the IL-8 pathway. *Sci Rep* 6**,** 18785.

Pozzobon, T., Facchinello, N., Bossi, F., Capitani, N., Benagiano, M., Di Benedetto, G., Zennaro, C., West, N., Codolo, G., Bernardini, M., Baldari, C.T., D'elios, M.M., Pellegrini, L., Argenton, F., and De Bernard, M. (2018). Corrigendum: Treponema pallidum (syphilis) antigen TpF1 induces angiogenesis through the activation of the IL-8 pathway. *Sci Rep* 8**,** 46945.

Prawiro, S.R., Poeranto, S., Amalia, A., Widyani, E.L., Indraswari, G., Soraya, M., Dwi Pradipto, S.R., Prasetya, A., Hidayat, G.R., and Alitha Putri, S.N. (2020). Generating mucosal and systemic immune response following vaccination of vibrio cholerae adhesion molecule against shigella flexneri infection. *Indian J Med Microbiol* 38**,** 37-45.

Radeff, J.M., Nagy, Z., and Stern, P.H. (2004). Rho and Rho kinase are involved in parathyroid hormone-stimulated protein kinase C alpha translocation and IL-6 promoter activity in osteoblastic cells. *J Bone Miner Res* 19**,** 1882-1891.

Raderer, M., Wrba, F., Kornek, G., Maca, T., Koller, D.Y., Weinlaender, G., Hejna, M., and Scheithauer, W. (1998). Association between Helicobacter pylori infection and pancreatic cancer. *Oncology* 55**,** 16-19.

Raga, S., Julia, M.R., Crespi, C., Figuerola, J., Martinez, N., Mila, J., and Matamoros, N. (2003). Gammadelta T lymphocytes from cystic fibrosis patients and healthy donors are high TNF-alpha and IFN-gamma-producers in response to Pseudomonas aeruginosa. *Respir Res* 4**,** 9.

Rapado-González, Ó., Majem, B., Muinelo-Romay, L., López-López, R., and Suarez-Cunqueiro, M.M. (2016). Cancer salivary biomarkers for tumours distant to the oral cavity. *International journal of molecular sciences* 17**,** 1531.

Rashid, H., Siddiqua, T.J., Hossain, B., Siddique, A., Kabir, M., Noor, Z., Alam, M., Ahmed, M., and Haque, R. (2021). MicroRNA Expression and Intestinal Permeability in Children Living in a Slum Area of Bangladesh. *Frontiers in molecular biosciences***,** 1237.

Rawla, P., Sunkara, T., and Gaduputi, V. (2019). Epidemiology of Pancreatic Cancer: Global Trends, Etiology and Risk Factors. *World J Oncol* 10**,** 10-27.

Reinis, A., Pilmane, M., Stunda, A., Vetra, J., Kroica, J., Rostoka, D., Salms, G., Vostroilovs, A., Dons, A., and Berzina-Cimdina, L. (2011). An in vitro and in vivo study on the intensity of adhesion and colonization by Staphylococcus epidermidis and Pseudomonas aeruginosa on originally synthesized biomaterials with different chemical composition and modified surfaces and their effect on expression of TNF-alpha, beta-defensin 2 and IL-10 in tissues. *Medicina (Kaunas)* 47**,** 560-565.

Reynolds, T.S., Courtney, C.M., Erickson, K.E., Wolfe, L.M., Chatterjee, A., Nagpal, P., and Gill, R.T. (2017). ROS mediated selection for increased NADPH availability in Escherichia coli. *Biotechnol Bioeng* 114**,** 2685-2689.

Rimessi, A., Bezzerri, V., Salvatori, F., Tamanini, A., Nigro, F., Dechecchi, M.C., Santangelo, A., Prandini, P., Munari, S., Provezza, L., Garreau De Loubresse, N., Muller, J., Ribeiro, C.M.P., Lippi, G., Gambari, R., Pinton, P., and Cabrini, G. (2018). PLCB3 Loss of Function Reduces Pseudomonas aeruginosa-Dependent IL-8 Release in Cystic Fibrosis. *Am J Respir Cell Mol Biol* 59**,** 428-436.

Riquelme, S.A., Lozano, C., Moustafa, A.M., Liimatta, K., Tomlinson, K.L., Britto, C., Khanal, S., Gill, S.K., Narechania, A., Azcona-Gutierrez, J.M., Dimango, E., Saenz, Y., Planet, P., and Prince, A. (2019). CFTR-PTEN-dependent mitochondrial metabolic dysfunction promotes Pseudomonas aeruginosa airway infection. *Sci Transl Med* 11.

Risch, H.A. (2003). Etiology of pancreatic cancer, with a hypothesis concerning the role of N-nitroso compounds and excess gastric acidity. *J Natl Cancer Inst* 95**,** 948-960.

Risch, H.A. (2012). Pancreatic cancer: Helicobacter pylori colonization, N-nitrosamine exposures, and ABO blood group. *Mol Carcinog* 51**,** 109-118.

Risch, H.A., Lu, L., Streicher, S.A., Wang, J., Zhang, W., Ni, Q., Kidd, M.S., Yu, H., and Gao, Y.T. (2017). Aspirin Use and Reduced Risk of Pancreatic Cancer. *Cancer Epidemiol Biomarkers Prev* 26**,** 68-74.

Risch, H.A., Lu, L., Wang, J., Zhang, W., Ni, Q., Gao, Y.T., and Yu, H. (2013). ABO blood group and risk of pancreatic cancer: a study in Shanghai and meta-analysis. *Am J Epidemiol* 177**,** 1326-1337.

Risch, H.A., Yu, H., Lu, L., and Kidd, M.S. (2010). ABO blood group, Helicobacter pylori seropositivity, and risk of pancreatic cancer: a case-control study. *J Natl Cancer Inst* 102**,** 502-505.

Rokad, F., Moseley, R., Hardy, R.S., Chukkapalli, S., Crean, S., Kesavalu, L., and Singhrao, S.K. (2017). Cerebral Oxidative Stress and Microvasculature Defects in TNF-alpha Expressing Transgenic and Porphyromonas gingivalis-Infected ApoE-/- Mice. *J Alzheimers Dis* 60**,** 359-369.

Rolston, K.V., Dholakia, N., Rodriguez, S., and Rubenstein, E.B. (1995). Nature and outcome of febrile episodes in patients with pancreatic and hepatobiliary cancer. *Support Care Cancer* 3**,** 414-417.

Romanova Iu, M., Tomova, A.S., Shingarova, L.N., Lunin, V.G., Kariagina, A.S., Lupu, I.P., and Gintsburg, A.L. (2008). [Mechanism of interaction of tumor necrosis factor (TNF-alpha) of macroorganism with the Salmonella enterica cells (ser. Typhimurium)]. *Mol Gen Mikrobiol Virusol***,** 18-22.

Romanova, Y.M., Scheglovitova, O.N., Boshnakov, R.H., Alekseeva, N.V., Stepanova, T.V., Tomova, A.S., and Gintsburg, A.L. (2002). TNF-alpha and gamma-irradiation induced activation of the Salmonella typhimurium reproduction in the organs of infected animals. *Russ J Immunol* 7**,** 129-134.

Romero-Castro, N.S., Vazquez-Villamar, M., Munoz-Valle, J.F., Reyes-Fernandez, S., Serna-Radilla, V.O., Garcia-Arellano, S., and Castro-Alarcon, N. (2020). Relationship between TNF-alpha, MMP-8, and MMP-9 levels in gingival crevicular fluid and the subgingival microbiota in periodontal disease. *Odontology* 108**,** 25-33.

Roussel, L., Lafayette, S., Nguyen, D., Baglole, C.J., and Rousseau, S. (2016). Differential Contribution of the Aryl-Hydrocarbon Receptor and Toll-Like Receptor Pathways to IL-8 Expression in Normal and Cystic Fibrosis Airway Epithelial Cells Exposed to Pseudomonas aeruginosa. *Front Cell Dev Biol* 4**,** 148.

Ruiz-Laguna, J., Prieto-Alamo, M.J., and Pueyo, C. (2000). Oxidative mutagenesis in Escherichia coli strains lacking ROS-scavenging enzymes and/or 8-oxoguanine defenses. *Environ Mol Mutagen* 35**,** 22-30.

Rupesh, K.R., Moushumi Priya, A., Prashanth, K., and Jayachandran, S. (2012). Inhibitory effects of bioactive leads isolated from Pseudomonas aeruginosa PS3 and Pseudomonas fluorescens PS7 on MAP kinases and down regulation of pro inflammatory cytokines (TNF-alpha, IL-1beta) and mediators (NO, iNOS and COX). *Toxicol In Vitro* 26**,** 571-578.

Ryz, N.R., Patterson, S.J., Zhang, Y., Ma, C., Huang, T., Bhinder, G., Wu, X., Chan, J., Glesby, A., Sham, H.P., Dutz, J.P., Levings, M.K., Jacobson, K., and Vallance, B.A. (2012). Active vitamin D (1,25-dihydroxyvitamin D3) increases host susceptibility to Citrobacter rodentium by suppressing mucosal Th17 responses. *Am J Physiol Gastrointest Liver Physiol* 303**,** G1299-1311.

Saade Lemus, P., Anderson, K., Smith, M., and Bullock, A. (2019). Spontaneous regression of pancreatic cancer with liver metastases. *BMJ Case Rep* 12.

Sakamoto, R., Kajihara, I., Mijiddorj, T., Otsuka-Maeda, S., Sawamura, S., Nishimura, Y., Kanemaru, H., Kanazawa-Yamada, S., Nakamura, K., Honda, N., Makino, K., Aoi, J., Igata, T., Makino, T., Masuguchi, S., Fukushima, S., Morinaga, J., Komohara, Y., and Ihn, H. (2021a). Existence of Staphylococcus aureus correlates with the progression of extramammary Paget's disease: potential involvement of interleukin-17 and M2-like macrophage polarization. *Eur J Dermatol* 31**,** 48-54.

Sakamoto, Y., Mima, K., Ishimoto, T., Ogata, Y., Imai, K., Miyamoto, Y., Akiyama, T., Daitoku, N., Hiyoshi, Y., and Iwatsuki, M. (2021b). Relationship between Fusobacterium nucleatum and antitumor immunity in colorectal cancer liver metastasis. *Cancer science* 112**,** 4470.

Sampaio Fernandes, M., Vaz, P., Braga, A.C., Sampaio Fernandes, J.C., and Figueiral, M.H. (2017). The role of IL-1 gene polymorphisms (IL1A, IL1B, and IL1RN) as a risk factor in unsuccessful implants retaining overdentures. *J Prosthodont Res* 61**,** 439-449.

Santos, J.C., Ladeira, M.S., Pedrazzoli, J., Jr., and Ribeiro, M.L. (2012). Relationship of IL-1 and TNF-alpha polymorphisms with Helicobacter pylori in gastric diseases in a Brazilian population. *Braz J Med Biol Res* 45**,** 811-817.

Satorres, S., Alcaraz, L., and Di Genaro, S. (2007). Association between high levels IL-8 and Staphylococcus aureus-specific IgA antibodies in subjects with type 1 diabetes mellitus from Argentina. *Diabetes Res Clin Pract* 77**,** 489-491.

Schulte, A., Pandeya, N., Fawcett, J., Fritschi, L., Risch, H.A., Webb, P.M., Whiteman, D.C., and Neale, R.E. (2015). Association between Helicobacter pylori and pancreatic cancer risk: a meta-analysis. *Cancer Causes Control* 26**,** 1027-1035.

Schulz, E., Schumann, M., Schneemann, M., Dony, V., Fromm, A., Nagel, O., Schulzke, J.-D., and Bücker, R. (2021). Escherichia coli Alpha-Hemolysin HlyA Induces Host Cell Polarity Changes, Epithelial Barrier Dysfunction and Cell Detachment in Human Colon Carcinoma Caco-2 Cell Model via PTEN-Dependent Dysregulation of Cell Junctions. *Toxins* 13**,** 520.

Schumann, J., Angermuller, S., Bang, R., Lohoff, M., and Tiegs, G. (1998). Acute hepatotoxicity of Pseudomonas aeruginosa exotoxin A in mice depends on T cells and TNF. *J Immunol* 161**,** 5745-5754.

Schumann, J., Bluethmann, H., and Tiegs, G. (2000). Synergism of Pseudomonas aeruginosa exotoxin A with endotoxin, superantigen, or TNF results in TNFR1- and TNFR2-dependent liver toxicity in mice. *Immunol Lett* 74**,** 165-172.

Semiramoth, N., Gleizes, A., Turbica, I., Sandre, C., Gorges, R., Kansau, I., Servin, A., and Chollet-Martin, S. (2009). Escherichia coli type 1 pili trigger late IL-8 production by neutrophil-like differentiated PLB-985 cells through a Src family kinase- and MAPK-dependent mechanism. *J Leukoc Biol* 85**,** 310-321.

Sethi, V., Vitiello, G.A., Saxena, D., Miller, G., and Dudeja, V. (2019). The Role of the Microbiome in Immunologic Development and its Implication For Pancreatic Cancer Immunotherapy. *Gastroenterology* 156**,** 2097-2115 e2092.

Shapira, L., Houri, Y., Barak, V., Soskolne, W.A., Halabi, A., and Stabholz, A. (1997). Tetracycline inhibits Porphyromonas gingivalis lipopolysaccharide-induced lesions in vivo and TNF alpha processing in vitro. *J Periodontal Res* 32**,** 183-188.

Shibata, J., Goto, H., Arisawa, T., Niwa, Y., Hayakawa, T., Nakayama, A., and Mori, N. (1999). Regulation of tumour necrosis factor (TNF) induced apoptosis by soluble TNF receptors in Helicobacter pylori infection. *Gut* 45**,** 24-31.

Shibata, T., Nagata, K., and Kobayashi, Y. (2010). The mechanism underlying the appearance of late apoptotic neutrophils and subsequent TNF-alpha production at a late stage during Staphylococcus aureus bioparticle-induced peritoneal inflammation in inducible NO synthase-deficient mice. *Biochim Biophys Acta* 1802**,** 1105-1111.

Shimamura, T., Fujisawa, T., Husain, S.R., Joshi, B., and Puri, R.K. (2010). Interleukin 13 mediates signal transduction through interleukin 13 receptor alpha2 in pancreatic ductal adenocarcinoma: role of IL-13 Pseudomonas exotoxin in pancreatic cancer therapy. *Clin Cancer Res* 16**,** 577-586.

Shionoya, K., Tonozuka, R., Itoi, T., Sofuni, A., Tsuchiya, T., Ishii, K., Tanaka, R., Mukai, S., Nagai, K., Yamamoto, K., and Nakamura, I. (2021). Severe Acute Cholangitis and Bacteremia Due to Campylobacter jejuni: A Case Report and Review of the Literature. *Intern Med* 60**,** 3737-3741.

Shirai, R., Kadota, J., Tomono, K., Ogawa, K., Iida, K., Kawakami, K., and Kohno, S. (1997). Protective effect of granulocyte colony-stimulating factor (G-CSF) in a granulocytopenic mouse model of Pseudomonas aeruginosa lung infection through enhanced phagocytosis and killing by alveolar macrophages through priming tumour necrosis factor-alpha (TNF-alpha) production. *Clin Exp Immunol* 109**,** 73-79.

Shrader, H.R., Miller, A.M., Tomanek-Chalkley, A., Mccarthy, A., Coleman, K.L., Ear, P.H., Mangalam, A.K., Salem, A.K., and Chan, C.H.F. (2021). Effect of bacterial contamination in bile on pancreatic cancer cell survival. *Surgery* 169**,** 617-622.

Singh, V., Pal, A., and Darokar, M.P. (2021). Glabridin synergy with norfloxacin induces ROS in multidrug resistant Staphylococcus aureus. *J Gen Appl Microbiol* 67**,** 269-272.

Siregar, G.A., Halim, S., and Sitepu, V.R. (2015). Serum TNF-a, IL-8, VEGF levels in Helicobacter pylori infection and their association with degree of gastritis. *Acta Med Indones* 47**,** 120-126.

Skerrett, S.J., Martin, T.R., Chi, E.Y., Peschon, J.J., Mohler, K.M., and Wilson, C.B. (1999). Role of the type 1 TNF receptor in lung inflammation after inhalation of endotoxin or Pseudomonas aeruginosa. *Am J Physiol* 276**,** L715-727.

Socransky, S.S., Haffajee, A.D., Smith, C., and Duff, G.W. (2000). Microbiological parameters associated with IL-1 gene polymorphisms in periodontitis patients. *J Clin Periodontol* 27**,** 810-818.

Sonoda, F., Oishi, K., Iwagaki, A., and Matsumoto, K. (1997). Endogenous tumor necrosis factor (TNF) alpha mediates neutrophil accumulation at the mid-phase of a murine model of Pseudomonas aeruginosa pneumonia. *Microbiol Immunol* 41**,** 601-608.

Staff, P.O. (2015). Correction: Correction: Induction of TLR-2 and TLR-5 Expression by Helicobacter pylori Switches cagPAI-Dependent Signalling Leading to the Secretion of IL-8 and TNF-alpha. *PLoS One* 10**,** e0144365.

Stasiewicz, M., Kwasniewski, M., and Karpinski, T.M. (2021). Microbial Associations with Pancreatic Cancer: A New Frontier in Biomarkers. *Cancers (Basel)* 13.

Staugas, R.E., Harvey, D.P., Ferrante, A., Nandoskar, M., and Allison, A.C. (1992). Induction of tumor necrosis factor (TNF) and interleukin-1 (IL-1) by Pseudomonas aeruginosa and exotoxin A-induced suppression of lymphoproliferation and TNF, lymphotoxin, gamma interferon, and IL-1 production in human leukocytes. *Infect Immun* 60**,** 3162-3168.

Steiner, T.S., Nataro, J.P., Poteet-Smith, C.E., Smith, J.A., and Guerrant, R.L. (2000). Enteroaggregative Escherichia coli expresses a novel flagellin that causes IL-8 release from intestinal epithelial cells. *J Clin Invest* 105**,** 1769-1777.

Stolzenberg-Solomon, R.Z., Blaser, M.J., Limburg, P.J., Perez-Perez, G., Taylor, P.R., Virtamo, J., Albanes, D., and Study, A. (2001). Helicobacter pylori seropositivity as a risk factor for pancreatic cancer. *J Natl Cancer Inst* 93**,** 937-941.

Stolzenberg-Solomon, R.Z., Dodd, K.W., Blaser, M.J., Virtamo, J., Taylor, P.R., and Albanes, D. (2003). Tooth loss, pancreatic cancer, and Helicobacter pylori. *Am J Clin Nutr* 78**,** 176-181.

Strickertsson, J.A., Desler, C., Martin-Bertelsen, T., Machado, A.M., Wadstrom, T., Winther, O., Rasmussen, L.J., and Friis-Hansen, L. (2013). Enterococcus faecalis infection causes inflammation, intracellular oxphos-independent ROS production, and DNA damage in human gastric cancer cells. *PLoS One* 8**,** e63147.

Suganuma, M., and Fujiki, H. (2005). [Mechanisms of action of tipa (TNF-alpha inducing protein), a new carcinogenic factor, released from H. pylori]. *Nihon Rinsho* 63 Suppl 11**,** 80-83.

Suganuma, M., Watanabe, T., Sueoka, E., Lim, I.K., and Fujiki, H. (2021). Role of TNF-alpha-Inducing Protein Secreted by Helicobacter pylori as a Tumor Promoter in Gastric Cancer and Emerging Preventive Strategies. *Toxins (Basel)* 13.

Suganuma, M., Watanabe, T., Yamaguchi, K., Takahashi, A., and Fujiki, H. (2012). Human gastric cancer development with TNF-alpha-inducing protein secreted from Helicobacter pylori. *Cancer Lett* 322**,** 133-138.

Suganuma, M., Yamaguchi, K., Ono, Y., Matsumoto, H., Hayashi, T., Ogawa, T., Imai, K., Kuzuhara, T., Nishizono, A., and Fujiki, H. (2008). TNF-alpha-inducing protein, a carcinogenic factor secreted from H. pylori, enters gastric cancer cells. *Int J Cancer* 123**,** 117-122.

Sultana, S., and Bishayi, B. (2020). Potential anti-arthritic and anti-inflammatory effects of TNF-alpha processing inhibitor-1 (TAPI-1): A new approach to the treatment of S. aureus arthritis. *Immunobiology* 225**,** 151887.

Sulzbach, D.E.O.H.S., Biolchi, V., Richardt Medeiros, H.R., Bizerra Gandor Jantsch, D.B., Knabben, D.E.O.B.D.L.K., Reckziegel, R., Goettert, M.I., Brum, I.S., and Pozzobon, A. (2016). Effect of Helicobacter pylori on NFKB1, p38alpha and TNF-alpha mRNA expression levels in human gastric mucosa. *Exp Ther Med* 11**,** 2365-2372.

Sumiyoshi, T., Uemura, K., Aoki, G., Kawano, R., Kitagawa, H., Kondo, N., Okada, K., Seo, S., Otsuka, H., and Takahashi, S. (2022). Increased clostridium difficile infection in the era of preoperative chemotherapy for pancreatic cancer. *Pancreatology* 22**,** 258-263.

Sun, C.-H., Li, B.-B., Wang, B., Zhao, J., Zhang, X.-Y., Li, T.-T., Li, W.-B., Tang, D., Qiu, M.-J., and Wang, X.-C. (2019). The role of Fusobacterium nucleatum in colorectal cancer: From carcinogenesis to clinical management. *Chronic diseases and translational medicine* 5**,** 178-187.

Sun, G., Liu, F., and Lin, T.J. (2005). Identification of Pseudomonas aeruginosa-induced genes in human mast cells using suppression subtractive hybridization: up-regulation of IL-8 and CCL4 production. *Clin Exp Immunol* 142**,** 199-205.

Sun, X., He, X., Tzipori, S., Gerhard, R., and Feng, H. (2009). Essential role of the glucosyltransferase activity in Clostridium difficile toxin-induced secretion of TNF-alpha by macrophages. *Microb Pathog* 46**,** 298-305.

Svedova, J., Tsurutani, N., Liu, W., Khanna, K.M., and Vella, A.T. (2016). TNF and CD28 Signaling Play Unique but Complementary Roles in the Systemic Recruitment of Innate Immune Cells after Staphylococcus aureus Enterotoxin A Inhalation. *J Immunol* 196**,** 4510-4521.

Tahara, T., Shibata, T., Yamashita, H., Yoshioka, D., Okubo, M., Yonemura, J., Kamiya, Y., Ishizuka, T., Nakagawa, Y., Nagasaka, M., Iwata, M., Nakamura, M., Hirata, I., and Arisawa, T. (2012). Synergistic effect of IL-1beta and TNF-alpha polymorphisms on the H. pylori-related gastric pre-malignant condition. *Hepatogastroenterology* 59**,** 2416-2420.

Tajima, H., Kitagawa, H., Tsukada, T., Nakanuma, S., Okamoto, K., Sakai, S., Makino, I., Furukawa, H., Nakamura, K., Hayashi, H., Oyama, K., Inokuchi, M., Nakagawara, H., Miyashita, T., Fujita, H., Itoh, H., Takamura, H., Ninomiya, I., Fushida, S., Fujimura, T., and Ohta, T. (2013). A phase I study of neoadjuvant chemotherapy with gemcitabine plus oral S-1 for resectable pancreatic cancer. *Mol Clin Oncol* 1**,** 768-772.

Takahashi, Y., Cueno, M.E., Kamio, N., Iinuma, T., Hasegawa, Y., and Imai, K. (2022). Porphyromonas gingivalis Mfa1 fimbria putatively binds to TLR2 and induces both IL-6 and IL-8 production in human bronchial epithelial cells. *Biochem Biophys Res Commun* 589**,** 35-40.

Takayama, S., Takahashi, H., Matsuo, Y., Okada, Y., and Manabe, T. (2007). Effects of Helicobacter pylori infection on human pancreatic cancer cell line. *Hepatogastroenterology* 54**,** 2387-2391.

Tamai, R., Deng, X., and Kiyoura, Y. (2009). Porphyromonas gingivalis with either Tannerella forsythia or Treponema denticola induces synergistic IL-6 production by murine macrophage-like J774.1 cells. *Anaerobe* 15**,** 87-90.

Tan, W., Duong, M.T., Zuo, C., Qin, Y., Zhang, Y., Guo, Y., Hong, Y., Zheng, J.H., and Min, J.J. (2022). Targeting of pancreatic cancer cells and stromal cells using engineered oncolytic Salmonella typhimurium. *Mol Ther* 30**,** 662-671.

Tang, B., Wang, K., Jia, Y.P., Zhu, P., Fang, Y., Zhang, Z.J., Mao, X.H., Li, Q., and Zeng, D.Z. (2016). Fusobacterium nucleatum-Induced Impairment of Autophagic Flux Enhances the Expression of Proinflammatory Cytokines via ROS in Caco-2 Cells. *PLoS One* 11**,** e0165701.

Tanimoto, Y., Arikawa, K., and Nishikawa, Y. (2013). Effect of diffusely adherent Escherichia coli strains isolated from diarrhoeal patients and healthy carriers on IL-8 secretion and tight junction barrier integrity of Caco-2 cells. *Vet Immunol Immunopathol* 152**,** 183-188.

Tavares, R., and Pathak, S.K. (2017). Helicobacter pylori Secreted Protein HP1286 Triggers Apoptosis in Macrophages via TNF-Independent and ERK MAPK-Dependent Pathways. *Front Cell Infect Microbiol* 7**,** 58.

Tavares, R., and Pathak, S.K. (2018a). Induction of TNF, CXCL8 and IL-1beta in macrophages by Helicobacter pylori secreted protein HP1173 occurs via MAP-kinases, NF-kappaB and AP-1 signaling pathways. *Microb Pathog* 125**,** 295-305.

Tavares, R., and Pathak, S.K. (2018b). Induction of TNF, CXCL8 and IL-1β in macrophages by Helicobacter pylori secreted protein HP1173 occurs via MAP-kinases, NF-κB and AP-1 signaling pathways. *Microbial pathogenesis* 125**,** 295-305.

Tawfeeq, M.H., and Nasir, H.M. (2018). Effects of Campylobacter jejuni infection on serum level of IL-6, IL-8 and TNF-α. *Journal of Pharmaceutical Sciences and Research* 10**,** 2049-2052.

Tikhonov, I., Doroshenko, T., Chaly, Y., Smolnikova, V., Pauza, C.D., and Voitenok, N. (2001). Down-regulation of CXCR1 and CXCR2 expression on human neutrophils upon activation of whole blood by S. aureus is mediated by TNF-alpha. *Clin Exp Immunol* 125**,** 414-422.

Tourani, M., Habibzadeh, M., Karkhah, A., Shokri-Shirvani, J., Barari, L., and Nouri, H.R. (2018). Association of TNF-alpha but not IL-1beta levels with the presence of Helicobacter pylori infection increased the risk of peptic ulcer development. *Cytokine* 110**,** 232-236.

Triantafillidis, J.K., Gikas, A., and Merikas, E. (2014). Treatment of inflammatory bowel disease patients with anti-TNF-alpha factors and immunosuppressives does not influence the prevalence of Helicobacter pylori infection. *Indian J Gastroenterol* 33**,** 383-384.

Trikudanathan, G., Philip, A., Dasanu, C.A., and Baker, W.L. (2011). Association between Helicobacter pylori infection and pancreatic cancer. A cumulative meta-analysis. *JOP* 12**,** 26-31.

Trindade, S.C., Olczak, T., Gomes-Filho, I.S., De Moura-Costa, L.F., Vale, V.C., Galdino-Neto, M., Alves Dos Santos, H., De Carvalho Filho, P.C., Stocker, A., Bendicho, M.T., Xavier, M.T., De Moraes Marcilio Cerqueira, E., and Meyer, R. (2013). Porphyromonas gingivalis HmuY-induced production of interleukin-6 and IL-6 polymorphism in chronic periodontitis. *J Periodontol* 84**,** 650-655.

Tsai, B.Y., Lai, Y.H., Chiu, C.W., Hsu, C.Y., Chen, Y.H., Chen, Y.L., Tsai, P.J., Hung, Y.P., and Ko, W.C. (2022). Effect of Doxycycline in Decreasing the Severity of Clostridioides difficile Infection in Mice. *Antibiotics (Basel)* 11.

Tsay, T.B., Yang, M.C., Chen, P.H., Lai, K.H., Huang, H.T., Hsu, C.M., and Chen, L.W. (2013). Blocking TNF-alpha enhances Pseudomonas aeruginosa-induced mortality in burn mice through induction of IL-1beta. *Cytokine* 63**,** 58-66.

Tseng, J., Do, J., Widdicombe, J.H., and Machen, T.E. (2006). Innate immune responses of human tracheal epithelium to Pseudomonas aeruginosa flagellin, TNF-alpha, and IL-1beta. *Am J Physiol Cell Physiol* 290**,** C678-690.

Tsuge, H., Tsurumura, T., Utsunomiya, H., Kise, D., Kuzuhara, T., Watanabe, T., Fujiki, H., and Suganuma, M. (2009). Structural basis for the Helicobacter pylori-carcinogenic TNF-alpha-inducing protein. *Biochem Biophys Res Commun* 388**,** 193-198.

Tsuruta, T., Inoue, R., Tsushima, T., Watanabe, T., Tsukahara, T., and Ushida, K. (2013). Oral Administration of EC-12 Increases the Baseline Gene Expression of Antiviral Cytokine Genes, IFN-gamma and TNF-alpha, in Splenocytes and Mesenteric Lymph Node Cells of Weaning Piglets. *Biosci Microbiota Food Health* 32**,** 123-128.

Tufano, M.A., Cipollaro De L'ero, G., Ianniello, R., Galdiero, M., and Galdiero, F. (1991). Protein A and other surface components of Staphylococcus aureus stimulate production of IL-1 alpha, IL-4, IL-6, TNF and IFN-gamma. *Eur Cytokine Netw* 2**,** 361-366.

Uchiyama, A.a.T., Lopes, M.S.M., Vana, M.N.M., and Peixoto, R.D. (2021). Clostridium difficile Infection Leading to Intestinal Pneumatosis in a Patient with a Recent Diagnosis of Pancreatic Cancer Local Recurrence: A Case Report and Literature Review. *Case Rep Oncol* 14**,** 1111-1117.

Urakawa, S., Yamasaki, M., Makino, T., Kurokawa, Y., Yamamoto, K., Goto, K., Haruna, M., Hirata, M., Morimoto-Okazawa, A., and Kawashima, A. (2021). The impact of ICOS+ regulatory T cells and Helicobacter pylori infection on the prognosis of patients with gastric and colorectal cancer: potential prognostic benefit of pre-operative eradication therapy. *Cancer Immunology, Immunotherapy* 70**,** 443-452.

Vallejo, J.G., Knuefermann, P., Mann, D.L., and Sivasubramanian, N. (2000). Group B Streptococcus induces TNF-alpha gene expression and activation of the transcription factors NF-kappa B and activator protein-1 in human cord blood monocytes. *J Immunol* 165**,** 419-425.

Van Diepen, A., Martina, C.A., Flierman, R., Janssen, R., and Van Dissel, J.T. (2007). Treatment with anti-TNF alpha does not induce reactivation of latent Salmonella enterica serovar Typhimurium infection in C3H/HeN mice. *Scand J Immunol* 65**,** 407-411.

Van Harten, R.M., Veldhuizen, E.J.A., Haagsman, H.P., and Scheenstra, M.R. (2022). The cathelicidin CATH-2 efficiently neutralizes LPS- and E. coli-induced activation of porcine bone marrow derived macrophages. *Vet Immunol Immunopathol* 244**,** 110369.

Vanhooren, V., Vandenbroucke, R.E., Dewaele, S., Van Hamme, E., Haigh, J.J., Hochepied, T., and Libert, C. (2013). Mice overexpressing beta-1,4-Galactosyltransferase I are resistant to TNF-induced inflammation and DSS-induced colitis. *PLoS One* 8**,** e79883.

Varley, C.D., Deodhar, A.A., Ehst, B.D., Bakke, A., Blauvelt, A., Vega, R., Yamashita, S., and Winthrop, K.L. (2014). Persistence of Staphylococcus aureus colonization among individuals with immune-mediated inflammatory diseases treated with TNF-alpha inhibitor therapy. *Rheumatology (Oxford)* 53**,** 332-337.

Venza, I., Cucinotta, M., Visalli, M., De Grazia, G., Oliva, S., and Teti, D. (2009). Pseudomonas aeruginosa induces interleukin-8 (IL-8) gene expression in human conjunctiva through the recruitment of both RelA and CCAAT/enhancer-binding protein beta to the IL-8 promoter. *J Biol Chem* 284**,** 4191-4199.

Verma, A.K., Bauer, C., Palani, S., Metzger, D.W., and Sun, K. (2021). IFN-gamma Drives TNF-alpha Hyperproduction and Lethal Lung Inflammation during Antibiotic Treatment of Postinfluenza Staphylococcus aureus Pneumonia. *J Immunol* 207**,** 1371-1376.

Wadstrom, T., Rydberg, J., Rozalska, B., and Lelwala-Guruge, J. (1994). Intravenous Helicobacter pylori induces low levels of TNF-alpha and IL-1 alpha in a murine model. *APMIS* 102**,** 49-52.

Wan, B., Zhang, Q., Ni, J., Li, S., Wen, D., Li, J., Xiao, H., He, P., Ou, H.Y., Tao, J., Teng, Q., Lu, J., Wu, W., and Yao, Y.F. (2017). Type VI secretion system contributes to Enterohemorrhagic Escherichia coli virulence by secreting catalase against host reactive oxygen species (ROS). *PLoS Pathog* 13**,** e1006246.

Wang, C., and Li, J. (2015). Pathogenic Microorganisms and Pancreatic Cancer. *Gastrointest Tumors* 2**,** 41-47.

Wang, H., Naseer, N., Chen, Y., Zhu, A.Y., Kuai, X., Galagedera, N., Liu, Z., and Zhu, J. (2017a). OxyR2 modulates OxyR1 activity and Vibrio cholerae oxidative stress response. *Infection and immunity* 85**,** e00929-00916.

Wang, H., Zhou, H., Duan, X., Jotwani, R., Vuddaraju, H., Liang, S., Scott, D.A., and Lamont, R.J. (2014a). Porphyromonas gingivalis-induced reactive oxygen species activate JAK2 and regulate production of inflammatory cytokines through c-Jun. *Infection and immunity* 82**,** 4118-4126.

Wang, H.Y., Lin, L., Fu, W., Yu, H.Y., Yu, N., Tan, L.S., Cheng, J.W., and Pan, Y.P. (2017b). Preventive effects of the novel antimicrobial peptide Nal-P-113 in a rat Periodontitis model by limiting the growth of Porphyromonas gingivalis and modulating IL-1beta and TNF-alpha production. *BMC Complement Altern Med* 17**,** 426.

Wang, J.F., Wang, J.B., Chen, H., Zhang, C.M., Liu, L., Pan, S.H., and Wu, C.J. (2008). Ultrasound-mediated microbubble destruction enhances gene transfection in pancreatic cancer cells. *Adv Ther* 25**,** 412-421.

Wang, P.L., Shirasu, S., Shinohar, M., Azuma, Y., Daito, M., Yasuda, H., and Ohura, K. (1999). IL-10 inhibits Porphyromonas gingivalis LPS-stimulated human gingival fibroblasts production of IL-6. *Biochem Biophys Res Commun* 263**,** 372-377.

Wang, S., Liu, K., Seneviratne, C.J., Li, X., Cheung, G.S., Jin, L., Chu, C.H., and Zhang, C. (2015). Lipoteichoic acid from an Enterococcus faecalis clinical strain promotes TNF-alpha expression through the NF-kappaB and p38 MAPK signaling pathways in differentiated THP-1 macrophages. *Biomed Rep* 3**,** 697-702.

Wang, S.X., Liu, Q.Y., and Li, Y. (2016). Lentinan ameliorates burn sepsis by attenuating CD4(+) CD25(+) Tregs. *Burns* 42**,** 1513-1521.

Wang, W., Park, C., Oh, E., Sung, Y., Lee, J., Park, K.H., and Kang, H. (2019). Benzophenone Compounds, from a Marine-Derived Strain of the Fungus Pestalotiopsis neglecta, Inhibit Proliferation of Pancreatic Cancer Cells by Targeting the MEK/ERK Pathway. *J Nat Prod* 82**,** 3357-3365.

Wang, X., Wang, S., Tang, X., Zhang, A., Grabinski, T., Guo, Z., Hudson, E., Berghuis, B., Webb, C., Zhao, P., and Cao, B. (2010). Development and evaluation of monoclonal antibodies against phosphatidylethanolamine binding protein 1 in pancreatic cancer patients. *J Immunol Methods* 362**,** 151-160.

Wang, X.M., Ma, Z.Y., and Song, N. (2018a). Inflammatory cytokines IL-6, IL-10, IL-13, TNF-alpha and peritoneal fluid flora were associated with infertility in patients with endometriosis. *Eur Rev Med Pharmacol Sci* 22**,** 2513-2518.

Wang, Y., Zhang, F.C., and Wang, Y.J. (2014b). Helicobacter pylori and pancreatic cancer risk: a meta- analysis based on 2,049 cases and 2,861 controls. *Asian Pac J Cancer Prev* 15**,** 4449-4454.

Wang, Y.N., Bheemanaboina, R.R.Y., Cai, G.X., and Zhou, C.H. (2018b). Novel purine benzimidazoles as antimicrobial agents by regulating ROS generation and targeting clinically resistant Staphylococcus aureus DNA groove. *Bioorg Med Chem Lett* 28**,** 1621-1628.

Wang, Z., Friedrich, C., Hagemann, S.C., Korte, W.H., Goharani, N., Cording, S., Eberl, G., Sparwasser, T., and Lochner, M. (2014c). Regulatory T cells promote a protective Th17-associated immune response to intestinal bacterial infection with C. rodentium. *Mucosal Immunol* 7**,** 1290-1301.

Warny, M., Keates, A.C., Keates, S., Castagliuolo, I., Zacks, J.K., Aboudola, S., Qamar, A., Pothoulakis, C., Lamont, J.T., and Kelly, C.P. (2000). p38 MAP kinase activation by Clostridium difficile toxin A mediates monocyte necrosis, IL-8 production, and enteritis. *J Clin Invest* 105**,** 1147-1156.

Watanabe, T., Takahashi, A., Suzuki, K., Kurusu-Kanno, M., Yamaguchi, K., Fujiki, H., and Suganuma, M. (2014). Epithelial-mesenchymal transition in human gastric cancer cell lines induced by TNF-alpha-inducing protein of Helicobacter pylori. *Int J Cancer* 134**,** 2373-2382.

Wei, A.L., Li, M., Li, G.Q., Wang, X., Hu, W.M., Li, Z.L., Yuan, J., Liu, H.Y., Zhou, L.L., Li, K., Li, A., and Fu, M.R. (2020a). Oral microbiome and pancreatic cancer. *World J Gastroenterol* 26**,** 7679-7692.

Wei, X., Zong, W., Gao, Y., Peng, S., Liu, K., and Zheng, Y. (2020b). Effects of the Traditional Chinese Medicine Tang Luo Ning on Intestinal Flora and Oxidative Stress in Diabetic Rats. *Evid Based Complement Alternat Med* 2020**,** 3452625.

Wen, B., Chen, J., Taibi, A., and Comelli, E. (2015). Citrobacter rodentium Infection Alters Murine Colonic microRNA Signature. *The FASEB Journal* 29**,** 252.253.

Wen, J., Wang, Y., Gao, C., Zhang, G., You, Q., Zhang, W., Zhang, Z., Wang, S., Peng, G., and Shen, L. (2018a). Helicobacter pylori infection promotes Aquaporin 3 expression via the ROS-HIF-1alpha-AQP3-ROS loop in stomach mucosa: a potential novel mechanism for cancer pathogenesis. *Oncogene* 37**,** 3549-3561.

Wen, S.H., Lin, L.N., Wu, H.J., Yu, L., Lin, L., Zhu, L.L., Li, H.Y., Zhang, H.L., and Li, C.C. (2018b). TNF-alpha increases Staphylococcus aureus-induced death of human alveolar epithelial cell line A549 associated with RIP3-mediated necroptosis. *Life Sci* 195**,** 81-86.

Wohrer, S., Hejna, M., and Raderer, M. (2003). Helicobacter pylori and pancreatic cancer. A working hypothesis from epidemiological studies. *JOP* 4**,** 163-164; author reply 164.

Woo, J.T., Nakagawa, H., Krecic, A.M., Nagai, K., Hamilton, A.D., Sebti, S.M., and Stern, P.H. (2005). Inhibitory effects of mevastatin and a geranylgeranyl transferase I inhibitor (GGTI-2166) on mononuclear osteoclast formation induced by receptor activator of NF kappa B ligand (RANKL) or tumor necrosis factor-alpha (TNF-alpha). *Biochem Pharmacol* 69**,** 87-95.

Wu, J.Y., Zhou, R.Y., Jiang, X., Wu, Q., and Zhang, H. (2012). [Effects of TNF-alpha, IFN-gamma on the ability of keratinocytes to kill intracellular Staphylococcus aureus]. *Sichuan Da Xue Xue Bao Yi Xue Ban* 43**,** 382-385.

Xia, X., Zhang, L., Wu, H., Chen, F., Liu, X., Xu, H., Cui, Y., Zhu, Q., Wang, M., Hao, H., Li, D.P., Fay, W.P., Martinez-Lemus, L.A., Hill, M.A., Xu, C., and Liu, Z. (2022). CagA(+) Helicobacter pylori, Not CagA(-) Helicobacter pylori, Infection Impairs Endothelial Function Through Exosomes-Mediated ROS Formation. *Front Cardiovasc Med* 9**,** 881372.

Xiao, H., Li, C., Jiang, Y., Li, R., and Xia, B. (2009). [The relationship among IL-10, TNF gene polymorphisms, Helicobacter pylori infection and gastroduodenal diseases in Hubei Han ethnic]. *Zhonghua Nei Ke Za Zhi* 48**,** 552-556.

Xiao, M., Wang, Y., and Gao, Y. (2013). Association between Helicobacter pylori infection and pancreatic cancer development: a meta-analysis. *PLoS One* 8**,** e75559.

Xie, C., Yi, J., Lu, J., Nie, M., Huang, M., Rong, J., Zhu, Z., Chen, J., Zhou, X., Li, B., Chen, H., Lu, N., and Shu, X. (2018). N-Acetylcysteine Reduces ROS-Mediated Oxidative DNA Damage and PI3K/Akt Pathway Activation Induced by Helicobacter pylori Infection. *Oxid Med Cell Longev* 2018**,** 1874985.

Xie, Y., Xu, M., Xiao, Y., Liu, Z., Jiang, C., Kuang, X., Wang, C., Wu, H., Peng, J., Li, C., Wang, Y., Liu, H., Liu, B., Zhang, X., Zhao, F., Zeng, T., Liu, S., and Wu, Y. (2017). Treponema pallidum flagellin FlaA2 induces IL-6 secretion in THP-1 cells via the Toll-like receptor 2 signaling pathway. *Mol Immunol* 81**,** 42-51.

Xu, B.F., Wang, Q.Q., Zhang, J.P., Hu, W.L., and Zhang, R.L. (2019). Treponema pallidum induces the activation of endothelial cells via macrophage-derived exosomes. *Arch Dermatol Res* 311**,** 121-130.

Xu, D., Mcsorley, S.J., Tetley, L., Chatfield, S., Dougan, G., Chan, W.L., Satoskar, A., David, J.R., and Liew, F.Y. (1998). Protective effect on Leishmania major infection of migration inhibitory factor, TNF-alpha, and IFN-gamma administered orally via attenuated Salmonella typhimurium. *J Immunol* 160**,** 1285-1289.

Xu, L., Zhan, W., Deng, Y., Liu, X., Gao, G., Sun, X., and Liang, G. (2022). ROS Turn Nanoparticle Fluorescence on for Imaging Staphylococcus aureus Infection in Vivo. *Adv Healthc Mater***,** e2200453.

Xu, Q., Gu, S., Chen, Y., Quan, J., Lv, L., Chen, D., Zheng, B., Xu, L., and Li, L. (2018). Protective effect of Pediococcus pentosaceus LI05 against Clostridium difficile infection in a mouse model. *Frontiers in microbiology* 9**,** 2396.

Xue, M.L., Willcox, M.D., Lloyd, A., Wakefield, D., and Thakur, A. (2001a). Regulatory role of IL-1beta in the expression of IL-6 and IL-8 in human corneal epithelial cells during Pseudomonas aeruginosa colonization. *Clin Exp Ophthalmol* 29**,** 171-174.

Xue, M.L., Zhu, H., Willcox, M., Wakefield, D., Lloyd, A., and Thakur, A. (2001b). The role of IL-1beta in the regulation of IL-8 and IL-6 in human corneal epithelial cells during Pseudomonas aeruginosa colonization. *Curr Eye Res* 23**,** 406-414.

Yadav, V.K., Singh, P.K., Kalia, M., Sharma, D., Singh, S.K., and Agarwal, V. (2018). Pseudomonas aeruginosa quorum sensing molecule N-3-oxo-dodecanoyl-l-homoserine lactone activates human platelets through intracellular calcium-mediated ROS generation. *Int J Med Microbiol* 308**,** 858-864.

Yam, C., Zhao, M., Hayashi, K., Ma, H., Kishimoto, H., Mcelroy, M., Bouvet, M., and Hoffman, R.M. (2010). Monotherapy with a tumor-targeting mutant of S. typhimurium inhibits liver metastasis in a mouse model of pancreatic cancer. *J Surg Res* 164**,** 248-255.

Yamaguchi, M., Nishimura, F., Naruishi, H., Soga, Y., Kokeguchi, S., and Takashiba, S. (2005). Thiazolidinedione (pioglitazone) blocks P. gingivalis- and F. nucleatum, but not E. coli, lipopolysaccharide (LPS)-induced interleukin-6 (IL-6) production in adipocytes. *J Dent Res* 84**,** 240-244.

Yamanoi, K., and Nakayama, J. (2018). Reduced alphaGlcNAc glycosylation on gastric gland mucin is a biomarker of malignant potential for gastric cancer, Barrett's adenocarcinoma, and pancreatic cancer. *Histochem Cell Biol* 149**,** 569-575.

Yamazaki, K., Polak, B., Bird, P.S., Gemmell, E., Hara, K., and Seymour, G.J. (1989). Effects of periodontopathic bacteria on IL-1 and IL-1 inhibitor production by human polymorphonuclear neutrophils. *Oral Microbiol Immunol* 4**,** 193-198.

Yan, X., Tien, B., Li, L., and Yuan, M. (1995). Cloning, expression and characterization of a single-chain antibody PS-9 targeted to pancreatic cancer. *Sci China B* 38**,** 1230-1236.

Yang, J., Wang, Q., Wang, C., Yang, R., Ahmed, M., Kumaran, S., Velu, P., and Li, B. (2020). Pseudomonas aeruginosa synthesized silver nanoparticles inhibit cell proliferation and induce ROS mediated apoptosis in thyroid cancer cell line (TPC1). *Artif Cells Nanomed Biotechnol* 48**,** 800-809.

Yang, J.S., Jeon, J.H., Jang, M.S., Kang, S.S., Ahn, K.B., Song, M., Yun, C.H., and Han, S.H. (2018). Vibrio cholerae OmpU induces IL-8 expression in human intestinal epithelial cells. *Mol Immunol* 93**,** 47-54.

Yang, T., Wang, R., Liu, H., Wang, L., Li, J., Wu, S., Chen, X., Yang, X., and Zhao, Y. (2021). Berberine regulates macrophage polarization through IL-4-STAT6 signaling pathway in Helicobacter pylori-induced chronic atrophic gastritis. *Life Sciences* 266**,** 118903.

Yang, Y., Wang, X., Moore, D.R., Lightfoot, S.A., and Huycke, M.M. (2012). TNF-alpha mediates macrophage-induced bystander effects through Netrin-1. *Cancer Res* 72**,** 5219-5229.

Yang, Y.W., Jiang, Y.Z., Hsu, C.M., and Chen, L.W. (2017). Pseudomonas aeruginosa Ventilator-Associated Pneumonia Induces Lung Injury through TNF-alpha/c-Jun NH2-Terminal Kinase Pathways. *PLoS One* 12**,** e0169267.

Yea, S.S., Yang, Y.I., Jang, W.H., Lee, Y.J., Bae, H.S., and Paik, K.H. (2001). Association between TNF-alpha promoter polymorphism and Helicobacter pylori cagA subtype infection. *J Clin Pathol* 54**,** 703-706.

Yee, M., Kim, S., Sethi, P., Duzgunes, N., and Konopka, K. (2014). Porphyromonas gingivalis stimulates IL-6 and IL-8 secretion in GMSM-K, HSC-3 and H413 oral epithelial cells. *Anaerobe* 28**,** 62-67.

Yeo, T.P., and Lowenfels, A.B. (2012). Demographics and epidemiology of pancreatic cancer. *Cancer J* 18**,** 477-484.

Yiemwattana, I., and Kaomongkolgit, R. (2015). Alpha-mangostin suppresses IL-6 and IL-8 expression in P. gingivalis LPS-stimulated human gingival fibroblasts. *Odontology* 103**,** 348-355.

Yoon, B.Y., Yeom, J.H., Kim, J.S., Um, S.H., Jo, I., Lee, K., Kim, Y.H., and Ha, N.C. (2014). Direct ROS scavenging activity of CueP from Salmonella enterica serovar Typhimurium. *Mol Cells* 37**,** 100-108.

Yoon, W.S., Chae, Y.S., Hong, J., and Park, Y.K. (2011). Antitumor therapeutic effects of a genetically engineered Salmonella typhimurium harboring TNF-alpha in mice. *Appl Microbiol Biotechnol* 89**,** 1807-1819.

Yoshimura, A., Hara, Y., Kaneko, T., and Kato, I. (1997). Secretion of IL-1 beta, TNF-alpha, IL-8 and IL-1ra by human polymorphonuclear leukocytes in response to lipopolysaccharides from periodontopathic bacteria. *J Periodontal Res* 32**,** 279-286.

Yu, G., Murphy, G., Michel, A., Weinstein, S.J., Mannisto, S., Albanes, D., Pawlita, M., and Stolzenberg-Solomon, R.Z. (2013). Seropositivity to Helicobacter pylori and risk of pancreatic cancer. *Cancer Epidemiol Biomarkers Prev* 22**,** 2416-2419.

Yu, X., Wang, L., Yao, C., Qiu, R., Cui, Y., Dai, D., Deng, J., Hou, G., Wang, Y., and Qian, J. (2017). MiR-21 Regulates Metabolic Adaptation of TH17 Cells during Autoimmunity and Host Defence. *bioRxiv***,** 218545.

Yui, S., Mikami, M., Mimaki, Y., Sashida, Y., and Yamazaki, M. (2001). [Inhibition effect of Amaryllidaceae alkaloids, lycorine and lycoricidinol on macrophage TNF-alpha production]. *Yakugaku Zasshi* 121**,** 167-171.

Yusuf, S., Soenarto, Y., Juffrie, M., and Lestariana, W. (2019). The effect of zinc supplementation on pro-inflammatory cytokines (TNF-α, IL-1 AND IL-6) in mice with Escherichia coli LPS-induced diarrhea. *Iranian Journal of Microbiology* 11**,** 412.

Zabaglia, L.M., Ferraz, M.A., Pereira, W.N., Orcini, W.A., De Labio, R.W., Neto, A.C., Wisnieski, F., De Oliveira, J.G., De Arruda Cardoso Smith, M., Payao, S.L., and Rasmussen, L.T. (2015). Lack of association among TNF-alpha gene expression, -308 polymorphism (G > A) and virulence markers of Helicobacter pylori. *J Venom Anim Toxins Incl Trop Dis* 21**,** 54.

Zabaglia, L.M., Sallas, M.L., Santos, M.P.D., Orcini, W.A., Peruquetti, R.L., Constantino, D.H., Chen, E., Smith, M.a.C., Payao, S.M., and Rasmussen, L.T. (2018). Expression of miRNA-146a, miRNA-155, IL-2, and TNF-alpha in inflammatory response to Helicobacter pylori infection associated with cancer progression. *Ann Hum Genet* 82**,** 135-142.

Zaga-Clavellina, V., Garcia-Lopez, G., Flores-Herrera, H., Espejel-Nunez, A., Flores-Pliego, A., Soriano-Becerril, D., Maida-Claros, R., Merchant-Larios, H., and Vadillo-Ortega, F. (2007). In vitro secretion profiles of interleukin (IL)-1beta, IL-6, IL-8, IL-10, and TNF alpha after selective infection with Escherichia coli in human fetal membranes. *Reprod Biol Endocrinol* 5**,** 46.

Zalatnai, A. (2003). Pancreatic cancer - a continuing challenge in oncology. *Pathol Oncol Res* 9**,** 252-263.

Zalewska-Ziob, M., Adamek, B., Strzelczyk, J.K., Gawron, K., Jarzab, B., Gubala, E., Kula, D., Krakowczyk, L., Sieron, A., and Wiczkowski, A. (2009). TNF-alpha expression in gastric mucosa of individuals infected with different virulent Helicobacter pylori strains. *Med Sci Monit* 15**,** BR166-171.

Zhang, A., Yu, Y., Liu, F., Wang, D., Zhou, H., and Zhang, F. (2013). [Effect of Staphylococcus aureus on the expressions of TLR2, IL-1beta, TNF-alpha and NF-kappaB in Bcap-37 cells]. *Xi Bao Yu Fen Zi Mian Yi Xue Za Zhi* 29**,** 488-491.

Zhang, J., Cui, H.H., Wang, Y., Zhang, Q.X., Deng, S.L., Chen, Y.D., and Chen, H. (2014). Study of Hgp44 from Porphyromonas gingivalis on inducing HUVECs to secrete IL-6 and IL-8. *Genet Mol Res* 13**,** 2208-2219.

Zhang, N., Liu, Y., Yuan, X., Liang, M., Wang, X., Wang, M., Kong, J., Yang, H., and Zhou, F. (2021a). Clinical significance of Fusobacterium nucleatum infection and regulatory T cell enrichment in esophageal squamous cell carcinoma. *Pathology and Oncology Research***,** 108.

Zhang, X., Borbet, T.C., Fallegger, A., Wipperman, M.F., Blaser, M.J., and Muller, A. (2021b). An Antibiotic-Impacted Microbiota Compromises the Development of Colonic Regulatory T Cells and Predisposes to Dysregulated Immune Responses. *mBio* 12.

Zhao, Y., Wang, J.W., Tanaka, T., Hosono, A., Ando, R., Tokudome, S., Soeripto, Triningsih, F.X., Triono, T., Sumoharjo, S., Achwan, E.Y., Gunawan, S., and Li, Y.M. (2013). Association between TNF-alpha and IL-1beta genotypes vs Helicobacter pylori infection in Indonesia. *World J Gastroenterol* 19**,** 8758-8763.

Zhou, J.H., Tang, B., Liu, X.L., He, D.W., and Yang, D.T. (2007). hTERT-targeted E. coli purine nucleoside phosphorylase gene/6-methylpurine deoxyribose therapy for pancreatic cancer. *Chin Med J (Engl)* 120**,** 1348-1352.

Zhu, H., Conibear, T.C., Thuruthyil, S.J., and Willcox, M.D. (2008). Pseudomonas aeruginosa quorum-sensing signal molecules induce IL-8 production by human corneal epithelial cells. *Eye Contact Lens* 34**,** 179-181.
